# Meiotic gene variants contribute to recurrent blastulation failure

**DOI:** 10.1093/hropen/hoag039

**Published:** 2026-05-07

**Authors:** Xueqin Chen, Lizhi Leng, Wenbin He, Weina Li, Lianyue Li, Xing Zhang, Xilin Xu, Jing Dai, Yifan Gu, Pingyuan Xie, Fei Meng, Huiling Hu, Miao Jin, Shujuan Ma, Fei Gong, Guangxiu Lu, Gang Liu, Yueqiu Tan, Ge Lin, Wei Zheng

**Affiliations:** NHC Key Laboratory of Human Stem Cell and Reproductive Engineering, Xiangya School of Basic Medical Sciences & Furong Laboratory, Central South University, Changsha, China; NHC Key Laboratory of Human Stem Cell and Reproductive Engineering, Xiangya School of Basic Medical Sciences & Furong Laboratory, Central South University, Changsha, China; Clinical Research Center for Reproduction and Genetics in Hunan Province, Reproductive and Genetic Hospital of CITIC-XIANGYA, Changsha, China; Clinical Research Center for Reproduction and Genetics in Hunan Province, Reproductive and Genetic Hospital of CITIC-XIANGYA, Changsha, China; Hunan Guangxiu Hospital, Hunan Normal University School of Medicine, Changsha, China; Clinical Research Center for Reproduction and Genetics in Hunan Province, Reproductive and Genetic Hospital of CITIC-XIANGYA, Changsha, China; Center for Medical Genetics, School of Life Sciences, Central South University, Changsha, China; Center for Medical Genetics, School of Life Sciences, Central South University, Changsha, China; Clinical Research Center for Reproduction and Genetics in Hunan Province, Reproductive and Genetic Hospital of CITIC-XIANGYA, Changsha, China; Hunan Guangxiu Hospital, Hunan Normal University School of Medicine, Changsha, China; NHC Key Laboratory of Human Stem Cell and Reproductive Engineering, Xiangya School of Basic Medical Sciences & Furong Laboratory, Central South University, Changsha, China; Clinical Research Center for Reproduction and Genetics in Hunan Province, Reproductive and Genetic Hospital of CITIC-XIANGYA, Changsha, China; NHC Key Laboratory of Human Stem Cell and Reproductive Engineering, Xiangya School of Basic Medical Sciences & Furong Laboratory, Central South University, Changsha, China; Clinical Research Center for Reproduction and Genetics in Hunan Province, Reproductive and Genetic Hospital of CITIC-XIANGYA, Changsha, China; Clinical Research Center for Reproduction and Genetics in Hunan Province, Reproductive and Genetic Hospital of CITIC-XIANGYA, Changsha, China; Hunan Guangxiu Hospital, Hunan Normal University School of Medicine, Changsha, China; Clinical Research Center for Reproduction and Genetics in Hunan Province, Reproductive and Genetic Hospital of CITIC-XIANGYA, Changsha, China; Clinical Research Center for Reproduction and Genetics in Hunan Province, Reproductive and Genetic Hospital of CITIC-XIANGYA, Changsha, China; Hunan Guangxiu Hospital, Hunan Normal University School of Medicine, Changsha, China; Clinical Research Center for Reproduction and Genetics in Hunan Province, Reproductive and Genetic Hospital of CITIC-XIANGYA, Changsha, China; Clinical Research Center for Reproduction and Genetics in Hunan Province, Reproductive and Genetic Hospital of CITIC-XIANGYA, Changsha, China; NHC Key Laboratory of Human Stem Cell and Reproductive Engineering, Xiangya School of Basic Medical Sciences & Furong Laboratory, Central South University, Changsha, China; Clinical Research Center for Reproduction and Genetics in Hunan Province, Reproductive and Genetic Hospital of CITIC-XIANGYA, Changsha, China; NHC Key Laboratory of Human Stem Cell and Reproductive Engineering, Xiangya School of Basic Medical Sciences & Furong Laboratory, Central South University, Changsha, China; Clinical Research Center for Reproduction and Genetics in Hunan Province, Reproductive and Genetic Hospital of CITIC-XIANGYA, Changsha, China; Hunan Guangxiu Hospital, Hunan Normal University School of Medicine, Changsha, China; NHC Key Laboratory of Human Stem Cell and Reproductive Engineering, Xiangya School of Basic Medical Sciences & Furong Laboratory, Central South University, Changsha, China; Clinical Research Center for Reproduction and Genetics in Hunan Province, Reproductive and Genetic Hospital of CITIC-XIANGYA, Changsha, China; NHC Key Laboratory of Human Stem Cell and Reproductive Engineering, Xiangya School of Basic Medical Sciences & Furong Laboratory, Central South University, Changsha, China; Clinical Research Center for Reproduction and Genetics in Hunan Province, Reproductive and Genetic Hospital of CITIC-XIANGYA, Changsha, China; NHC Key Laboratory of Human Stem Cell and Reproductive Engineering, Xiangya School of Basic Medical Sciences & Furong Laboratory, Central South University, Changsha, China; Clinical Research Center for Reproduction and Genetics in Hunan Province, Reproductive and Genetic Hospital of CITIC-XIANGYA, Changsha, China; Hunan Guangxiu Hospital, Hunan Normal University School of Medicine, Changsha, China; NHC Key Laboratory of Human Stem Cell and Reproductive Engineering, Xiangya School of Basic Medical Sciences & Furong Laboratory, Central South University, Changsha, China; Clinical Research Center for Reproduction and Genetics in Hunan Province, Reproductive and Genetic Hospital of CITIC-XIANGYA, Changsha, China; Center for Medical Genetics, School of Life Sciences, Central South University, Changsha, China

**Keywords:** early embryonic arrest, mutation, blastulation-failure, aneuploid, infertility

## Abstract

**STUDY QUESTION:**

What is the genetic etiology of recurrent blastulation failure, particularly in morphologically good-quality cleavage-stage embryos?

**SUMMARY ANSWER:**

Variants in meiotic genes may contribute to gamete-derived complex aneuploidy and impaired embryonic genome activation, which are strongly associated with recurrent blastulation failure in good-quality cleavage-stage embryos (R-GQBF).

**WHAT IS KNOWN ALREADY:**

Successful blastocyst formation is critical for implantation. Embryonic development undergoes a major transition around the 8‑cell stage, shifting from reliance on maternal transcripts to embryonic genome activation. While pathogenic variants in maternal-effect genes have been linked to developmental arrest before the 8-cell stage, the genetic basis of failure occurring between the 8-cell stage and blastulation remains unclear.

**STUDY DESIGN, SIZE, DURATION:**

From 2018 to 2023, 707 couples who met the R-GQBF criteria were recruited. After rigorous exclusion, 204 couples remained, of whom 97 were ultimately included for genetic etiology analysis.

**PARTICIPANTS/MATERIALS, SETTING, METHODS:**

A total of 109 individuals from 97 selected couples (93 females and 16 males, including 13 couples with both partners) underwent whole-exome sequencing (WES). Genetic variants were compared with those from 1000 fertile controls (500 females and 500 males). The chromosomal constitutions of 103 blastulation-failure embryos from 50 R-GQBF couples were analyzed via WES. Copy number variation (CNV) parental origin analysis was performed on 13 aneuploid embryos. Paternally derived chromosomal anomalies were further investigated using single-sperm chromosome analysis. Additionally, single-cell RNA sequencing was carried out on 15 arrested embryos.

**MAIN RESULTS AND THE ROLE OF CHANCE:**

Twenty-five variants in 10 meiotic genes were identified in 20 patients (18.3%). Female carriers predominantly harbored prophase I variants (*SPO11*, *MEI1*, *REC114*, *ANKRD31*, *DMC1*, *CNTD1*, *MLH3*, *SYCE1*, and *SYCP2*), while three male patients carried *MEIKIN* variants. Female carriers generally had preserved ovarian reserve, whereas male carriers showed severe oligoasthenoteratozoospermia. Chromosomal analysis revealed a high prevalence of complex aneuploidy in blastulation-failure embryos (63.1%). CNV tracing confirmed the parental origin of abnormalities from the meiotic variant carrier in analyzed case, and sperm from all *MEIKIN* carriers also exhibited severe chromosomal abnormalities. Single-cell transcriptomics revealed defective embryonic genome activation, impaired lineage specification, and activation of stress pathways. These consistent findings minimize the likelihood of chance associations.

**LARGE SCALE DATA:**

N/A.

**LIMITATIONS, REASONS FOR CAUTION:**

Direct evaluation of oocyte chromosomal integrity was not feasible. Larger cohorts may reveal additional genes, and functional studies in animal models are required to validate genotype–phenotype relationships.

**WIDER IMPLICATIONS OF THE FINDINGS:**

This study identifies biparental meiotic variants as an underrecognized cause of R-GQBF. Clinically, beyond routine extended embryo culture, chromosomal analysis should be recommended for etiological investigation. For couples experiencing recurrent complex aneuploidy in good-quality cleavage-stage embryos, genetic screening for meiotic variants may aid in diagnosis, counseling, and personalized treatment planning.

**STUDY FUNDING/COMPETING INTEREST(S):**

This work was supported by the National Natural Science Foundation of China (32270911, 82371672, 82471697, 62588301), the Natural Science Foundation of Hunan Province (2024JJ2083, 2023JJ30714, 2023JJ10084), the Science and Technology Innovation Program of Hunan Province (2023RC3233), the Fundamental and Interdisciplinary Disciplines Breakthrough Plan of the Ministry of Education of China (JYB2025XDXM117), the National Key R&D Program of China (2023YFC2705504), and the project of the Reproductive and Genetic Hospital of CITIC-XIANGYA (YNXM-202221, YNXM-202402, and YNXM-202505). The authors declare no competing interests.

WHAT DOES THIS MEAN FOR PATIENTS? For a pregnancy to succeed, an embryo must have the potential to develop into a tiny hollow ball of cells (called a blastocyst) before it can successfully implant in the uterus. However, some patients experience repeated failure at this stage, even when their early embryos appear normal under a routine microscope check.

In this study, we investigated couples whose embryos initially appear normal but consistently fail to reach this stage. We found that, in some cases, this may be linked to changes in genes that are important for the formation of sperm and eggs. These genetic changes can lead to severe errors in how chromosomes (the packages of DNA) are divided when sperm and eggs are formed. Such problems may not be detected by the embryo’s early quality-control systems, but can later stop the embryo from developing further.

Our findings suggest that potential genetic factors inherited from the parents can contribute to this type of repeated failure. This may help explain why some patients do not achieve a successful pregnancy even though their early embryos appear healthy. In the future, genetic testing might help doctors better understand such cases and offer more personalized advice and treatment options.

## Introduction

Infertility, which affects an estimated 12.6–17.5% of couples globally, is projected to become the third most prevalent health challenge of the 21st century (Cox *et al.*, 2022; Liu *et al.*, 2022). ART represents one of the most effective treatments, yet its success rates have plateaued at ∼30–40% (Kushnir *et al.*, 2017; Doulgeraki and Iliodromiti, 2023). A critical determinant of ART outcome is the embryo’s inherent developmental potential—in particular, its capacity to progress to the blastocyst stage, which is essential for successful uterine implantation (Zhang *et al.*, 2013a).

Preimplantation development proceeds through two major, functionally distinct phases. Initially, the embryo relies on maternally derived transcripts and proteins to complete the early cleavages up to the 8‑cell stage (Eckersley-Maslin *et al.*, 2018). This is followed by a pivotal developmental transition marked by embryonic genome activation (EGA), which shifts transcriptional control from the oocyte to the embryo and enables subsequent differentiation and blastocyst formation. The 8‑cell stage thus serves as a critical checkpoint, representing the shift from maternal to zygotic genetic control (Jukam *et al.*, 2017; Kojima *et al.*, 2025).

Although pathogenic variants in maternal‑effect genes are known to cause arrest prior to the 8‑cell stage (Zhang *et al.*, 2026), the genetic basis of developmental failure occurring specifically between the 8‑cell stage and blastulation remains poorly understood. Previous studies, including our own work linking defective zygotic decay to 8‑cell arrest, have highlighted the sensitivity of this transition window (Sha *et al.*, 2020). Other factors such as epigenetic dysregulation, cytoskeletal abnormalities, and metabolic defects have also been implicated in sporadic blastulation impairment (Yang *et al.*, 2022; Hernandez Mora *et al.*, 2023; Wang *et al.*, 2024). However, a systematic genetic etiology for recurrent blastulation failure following morphologically normal early cleavage has not been established.

Here, we delineate a distinct subtype of preimplantation developmental arrest within the broader spectrum of preimplantation embryonic lethality (PREMBL) (Alazami *et al.*, 2015), termed recurrent good-quality cleavage-stage blastulation failure (R-GQBF). This subtype is characterized by apparently normal early cleavages, resulting in morphologically good-quality 6–8 cell embryos by Day 3, which then consistently fail to form viable blastocysts during extended *in vitro* culture. To explore its genetic basis, we performed whole-exome sequencing (WES) in a rigorously selected cohort and identified pathogenic variants in 10 meiotic genes across both female and male patients. We further indicated that meiotic chromosome segregation errors in gametes are associated with complex aneuploidy in analyzed embryos. Transcriptomic profiling of arrested embryos suggested that subsequent impaired EGA and hyperactivation of stress pathways may contributed to this developmental arrest. Collectively, our findings point to biparental meiotic variants may a potential genetic etiology for R‑GQBF and support the integration of targeted genetic analysis into the diagnostic evaluation of patients with unexplained recurrent blastulation failure.

## Materials and methods

### Study subjects

Probands diagnosed with R-GQBF were recruited from the Reproductive and Genetic Hospital of CITIC-XIANGYA. All participants provided written informed consent prior to enrollment. The study was conducted in accordance with the principles outlined in the Declaration of Helsinki. Ethical approval was obtained from the Ethics Committee of the Reproductive and Genetic Hospital of CITIC-XIANGYA (Approval No. LL-SC-2017-012 and LL-SC-2024-012).

### Definition and assessment of embryos

Cleavage-stage embryos were defined based on the Istanbul Consensus (Working Group on the update of the ESHRE/ALPHA Istanbul Consensus *et al.*, 2025), in alignment with Chinese expert consensus and clinical practice, the good-quality cleavage-stage embryo was defined as a normally fertilized (2PN) embryo at ∼68-h post-fertilization, with ≥6 cells, ≤20% fragmentation, uniform blastomeres, and no multinucleation. The good-quality cleavage-stage embryo rate was defined as the number of good-quality cleavage-stage embryos divided by the number of 2PN embryos. Blastocysts were evaluated according to the Gardner scoring system (Gardner and Schoolcraft, 1999), and those graded ≥ 4BC were considered suitable for transfer. The blastocyst formation rate was defined as the number of transferable blastocysts divided by the number of good-quality cleavage-stage embryos cultured.

### Whole-exome sequencing and variant analysis

Genomic DNA was extracted from peripheral blood samples using standard procedures (Qiagen, Hilden, Germany, 51106). Whole-exome capture followed by sequencing was conducted on the Illumina NovaSeq 6000 platform. Raw sequencing reads (FASTQ files) were aligned to the human reference genome (GRCh37), the variant calling and processed following the Genome Analysis Toolkit (GATK) best‑practices workflow implemented in Sentieon Genomics software (v20211202) (Sentieon Inc., Can Jose, CA, USA) (McKenna *et al.*, 2010). The resulting variant call sets were subsequently annotated with Annovar (v20200608) based on the RefSeq gene model (GRCh37/hg19). Annotated variants filtered according to established criteria (Zheng *et al.*, 2020; Dai *et al.*, 2021): (i) Population frequency filtering: minor allele frequencies (frequencies < 1% in the genome aggregation database (gnomAD) (v2.1.1) and Exome Aggregation Consortium (ExAC) (v1.0) databases); (ii) Functional impact filtering: variants including coding insertions/deletions (indels), non-synonymous single-nucleotide variants, and splice site alterations; and (iii) 4087 genes demonstrating documented high or specific expression in gamete and prior associations with human infertility. This gene set was curated by integrating gamete-elevated genes from the Human Protein Atlas (https://www.proteinatlas.org) (Fagerberg *et al.*, 2014; Uhlen *et al.*, 2015; Guo *et al.*, 2018; Wagner *et al.*, 2020) and meiosis‑related genes with functional annotations or supported by mouse knockout studies (Kim *et al.*, 2015; Gopinathan *et al.*, 2017; Yueh *et al.*, 2021; Li *et al.*, 2022; Xie *et al.*, 2022b). The complete gene list is provided in Supplementary Table S1.

Candidate variants identified by WES were validated using Sanger sequencing, with segregation analysis performed in all 20 available pedigrees. Family members (parents and siblings, where accessible) were recruited to assess inheritance patterns based on three models: autosomal recessive (homozygous or compound heterozygous), autosomal dominant, and X‑linked inheritance. A variant was considered consistent if it co‑segregated with the infertility phenotype according to the expected model. Primers were designed to flank each variant site.

### Semen parameter analysis

Semen samples were collected after 2–7 days of abstinence and analyzed using a computer-assisted sperm analyzer (Saisi Medical Technology (Beijing) Co., Ltd., Beijing, China) and Papanicolaou staining according to WHO (5th edition) guidelines, with morphology assessed on at least 200 sperm per sample.

### Toluidine blue staining

Sperm chromatin integrity was assessed using a toluidine blue staining test kit (Leagene Biotechnology (Beijing) Co., Ltd., Beijing, China, DA1201) following the manufacturer’s protocol. Briefly, washed sperm were smeared onto glass slides, air-dried, and fixed in fixation solution for 90 s. Slides were then stained with toluidine blue working solution for 5 min, rinsed in distilled water, decolorized in elution buffer for 20 min, air-dried, and were mounted with coverslips. At least 200 sperm were evaluated per sample by light microscopy and categorized based on nuclear staining intensity as either pale blue (indicative of normal chromatin condensation) or dark blue to violet (indicative of abnormal chromatin condensation). The control sample was obtained from a 30-year-old sperm donor with confirmed fertility, normal semen parameters, and exclusion of *MEIKIN* variants.

### Sperm fluorescence *in situ* hybridization

Sperm fluorescence *in situ* hybridization (FISH) was performed as previously described (Cheng *et al.*, 2012). Briefly, sperm were washed in PBS, fixed in methanol: acetic acid (3:1) for 30 min, and spread onto slides. After drying at 60°C, slides underwent chromatin decondensation (0.25 M DTT, 30 min, 25°C), RNase A treatment (100 μg/ml in 2× saline–sodium citrate buffer (SSC), 1 h, 37°C), and DNA denaturation (70% formamide/2× SSC, 5 min, 75°C). Multiplex FISH was performed using Vysis TelVysion centromeric probes (Abbott Molecular Inc., Des Plaines, IL, USA) targeting chromosomes 1, 2, 3, 4, 6, 7, 9, 12, 15, 18, X, and Y. Probes were denatured at 75°C for 7 min and applied to slides (3 μl per sample), followed by hybridization at 37°C for 16 h in a humidified chamber. Post-hybridization washes were conducted in 0.4× SSC/0.3% NP-40 at 45°C for 2 min and 2× SSC/0.1% NP-40 at room temperature for 1 min. Nuclei were counterstained with DAPI and analyzed using fluorescence microscopy. Only morphologically intact sperm nuclei with clearly distinguishable, non-overlapping FISH signals and adequate DAPI staining were included. Disomy was defined as two signals for a given chromosome, nullisomy as absence of signal, and diploidy as two signals for each tested chromosome. Abnormality frequencies were calculated as percentages of total analyzable sperm nuclei. The control sample was obtained from a 30-year-old sperm donor with confirmed fertility, normal semen parameters, and exclusion of *MEIKIN* variants.

### Single-sperm whole-genome amplification and sequencing

Single-sperm isolation was performed using ICSI micropipettes integrated with a Nikon Diaphot micromanipulation system. Whole-genome amplification (WGA) of single sperm was conducted using the multiple displacement amplification (MDA) protocol (Qiagen, 150343) following the manufacturer’s instructions.

For initial chromosomal profiling, low-coverage whole-genome sequencing (∼5 million 150-bp single-end reads per sample) was performed on the DA8600 platform (Basecare Medical, Suzhou, China). Reads were aligned to GRCh37 using BWA-MEM (v0.7.17), binned into 20-kb windows, and GC-normalized. Copy number variations (CNVs) were inferred relative to the haploid reference, where an increase from 1 to 2 copies indicated gain, and a decrease from 1 to 0 indicated loss.

High-resolution ploidy determination was conducted using haplotyping-based preimplantation genetic testing (HaploPGT) (Xie *et al.*, 2022a). Amplified libraries were sequenced on the DA500/DA5000 platform (Basecare) to ≥80 million 100-bp paired-end reads per sample. Data analysis included the following steps: (i) alignment to GRCh37 using BWA-MEM; (ii) binning of reads into 10-kb windows; (iii) GC-content normalization via LOESS regression; and (iv) calibration against diploid control datasets.

Ploidy status was inferred by integrating two parameters: CNV profiles and the heterozygosity index (HI), defined as log_2_(*R*_sample_/*R*_diploid_), where *R*_sample_ and *R*_diploid_ represent the heterozygosity rates in the test sperm and diploid reference samples, respectively. Interpretation criteria were defined as: HI≈−1 (haploid, genome-wide homozygosity), HI ≈ 0 (diploid, balanced heterozygosity), or regional hetero-/homozygosity patterns (potential diploidy).

The control sample was obtained from a 30-year-old sperm donor with confirmed fertility, normal semen parameters, and exclusion of *MEIKIN* variants.

### Collection of blastulation-failure embryos

Developmental arrest was defined as the absence of cleavage or morphological progression over a subsequent 24-h period, according to Istanbul Consensus Criteria (Working Group on the update of the ESHRE/ALPHA Istanbul Consensus *et al.*, 2025). To exclude apoptotic or degenerating embryos, samples were collected immediately upon arrest confirmation and only if they displayed preserved morphology, characterized by intact cell membranes, homogeneous cytoplasm without darkening, ≤20% fragmentation, and no signs of cellular lysis. Before processing, the zona pellucida was removed using Tyrode’s solution (Sigma-Aldrich, St. Louis, MO, USA, T1788) to avoid somatic cell contamination, followed by three washes in 1% bovine serum albumin (Sigma-Aldrich, A1933).

### WGA and sequencing of blastulation-failure embryos

Genomic DNA from blastulation-failure embryos was extracted and amplified using the PicoPLEX WGA Kit (Takara Biomedical Technology (Beijing) Co., Ltd., Beijing, China, R300672) according to the manufacturer’s instructions. Low-coverage whole-genome sequencing was performed on the DA500 platform (Basecare), which generated ∼10 million 100-bp single-end reads per embryo. Reads were aligned to the GRCh37 human reference genome, and CNV analysis was performed using the BasePGT-A (Basecare), as described previously (Zhang *et al.*, 2013b). Embryos were classified as euploid if no significant deviations from diploid baseline were observed. Mosaicism was defined as CNVs affecting 30–70% of cells, while aneuploidy was diagnosed in embryos with >70% abnormal cells; embryos with <30% abnormal cells were considered euploid.

### Haplotyping-based preimplantation genetic testing and CNV origin analysis

Parental origin of chromosomal abnormalities in embryos with complex aneuploidy was determined using HaploPGT (Xie *et al.*, 2022a). Parental genomic DNA was extracted from peripheral blood, and amplified embryonic DNA was sequenced (100-bp paired-end, ≥80 million reads/sample) on the DA500 platform. Reads were aligned to GRCh37, binned into 10-kb intervals, normalized for GC content via LOESS regression, and calibrated against diploid reference genomes.

In CNV origin analysis, we identified informative single-nucleotide polymorphism (SNP) patterns characterized by paternal homozygous AA and maternal homozygous BB loci (designated as AABB) or the reciprocal pattern (BBAA). The B-allele frequency (BAF) and genotype distribution (AA, BB, AB) within these loci enabled discrimination of CNV types. A *Z*-test comparing BAF values between CNV and non-CNV regions (theoretical BAF ≈ 0.5 in diploid regions) identified significant deviations (|*Z*-score| > 3). Maternal duplication was inferred when AABB-AB-BAF > 0.5 with concurrent BBAA-AB-BAF < 0.5, whereas paternal duplication was indicated by reversed trends (AABB-AB-BAF < 0.5 and BBAA-AB-BAF > 0.5). Chromosomal loss was defined by genotype ratios: paternal copy loss (AABB-BB-ratio > 0.8) or maternal copy loss (AABB-AA-ratio > 0.8) in embryos with parental AABB genotypes.

### Single-cell RNA-seq library preparation, sequencing, and data analysis

RNA-seq libraries were prepared using the SMART-Seq2 protocol as previously described (Leng *et al.*, 2019). Each library was generated from a single arrested embryo, with each embryo treated as one independent sample rather than pooled material. Libraries were sequenced on the BGISEQ-500 platform (BGI-Shenzhen, Shenzhen, China) to generate 100-bp paired-end reads.

Raw data were processed with Fastp (v0.20.0) for adapter trimming and quality filtering (Chen *et al.*, 2018). Clean reads were aligned to the GRCh38 reference genome (unmasked) using STAR (v2.7.8a) (Dobin *et al.*, 2013). Gene-level read counts were generated using StringTie (v2.0.4) (Pertea *et al.*, 2015). Although transcript abundance was also normalized as transcripts per million (TPM) for expression visualization and filtering, differential expression analysis was performed using raw gene-level count matrices as input to the DESeq2 R package (Love *et al.*, 2014). Given the full-length SMART-Seq2 protocol and embryo-to-embryo variability, each embryo was analyzed as an individual biological replicate, and normalization and dispersion estimation were performed within DESeq2 using its internal size-factor normalization and variance modeling framework to account for variability inherent to SMART-Seq2 datasets. Differentially expressed genes (DEGs) were identified using the DESeq2 R package, with the selection criteria of *q*-value ≤ 0.05, fold change ≥ 2, and TPM ≥ 1 in more than half of the samples within a given group. Gene ontology (GO) enrichment analysis of DEGs was conducted using Metascape (Zhou *et al.*, 2019) (http://metascape.org), focusing on biological process terms.

### Statistical analysis

The results are presented as mean ± SEM. Statistical comparisons between sperm and embryo groups were performed using two-tailed unpaired Student’s *t*-tests. Analyses were conducted using Microsoft Excel (Microsoft Corporation, Redmond, WA, USA). Values of *P* < 0.05 were considered statistically significant.

## Results

### Meiotic variants identified in R-GQBF couples

The assessment of cleavage‑stage embryos and transfer‑ready blastocysts followed the Istanbul consensus and Gardner scoring system, respectively, as detailed in the Materials and methods section. We first recruited a cohort of 707 couples meeting the R-GQBF criteria, which required recurrent blastulation failure and/or implantation failure, as well as a blastocyst formation rate of ≤ 20% (corresponding to the lowest 10th percentile of our internal dataset of 47 093 eligible cycles) across at least two independent ART cycles, despite adequate cleavage-stage embryo quality (≥ 40% good-quality cleavage-stage embryos per cycle, which is close to the median of 45.45% in our internal cohort of 68 780 blastocyst culture cycles).

**Figure 1.**
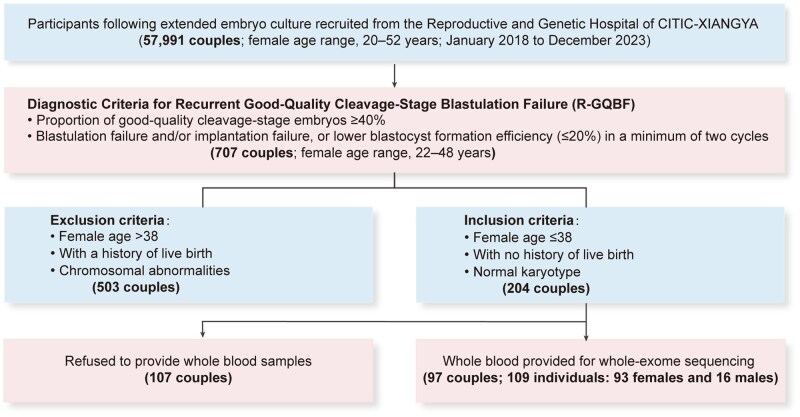
**Genetic screening workflow for recurrent good-quality cleavage-stage blastulation failure (R-GQBF)**. Workflow for cohort selection. A total of 707 couples with R-GQBF were initially screened. After excluding cases with advanced reproductive age, chromosomal abnormalities, or a history of live birth, whole-exome sequencing (WES) was finally performed on 97 eligible couples, including 93 females and 16 males.

To investigate the genetic underpinnings of R-GQBF, we applied stringent exclusion criteria to eliminate cases involving advanced reproductive age (>38 years), carriers of chromosomal abnormalities, and a history of live birth, resulting in a final study population of 204 eligible couples. WES was subsequently performed on genomic DNA from 97 of these couples, corresponding to 109 individuals (93 females, 16 males, with both partners sequenced in 13 couples; Fig. 1). WES data from 1000 unrelated, proven fertile individuals (500 males and 500 females confirmed natural conception) were used as population controls. Variant annotation and filtering were conducted as described in the Materials and methods section.

**Figure 2.**
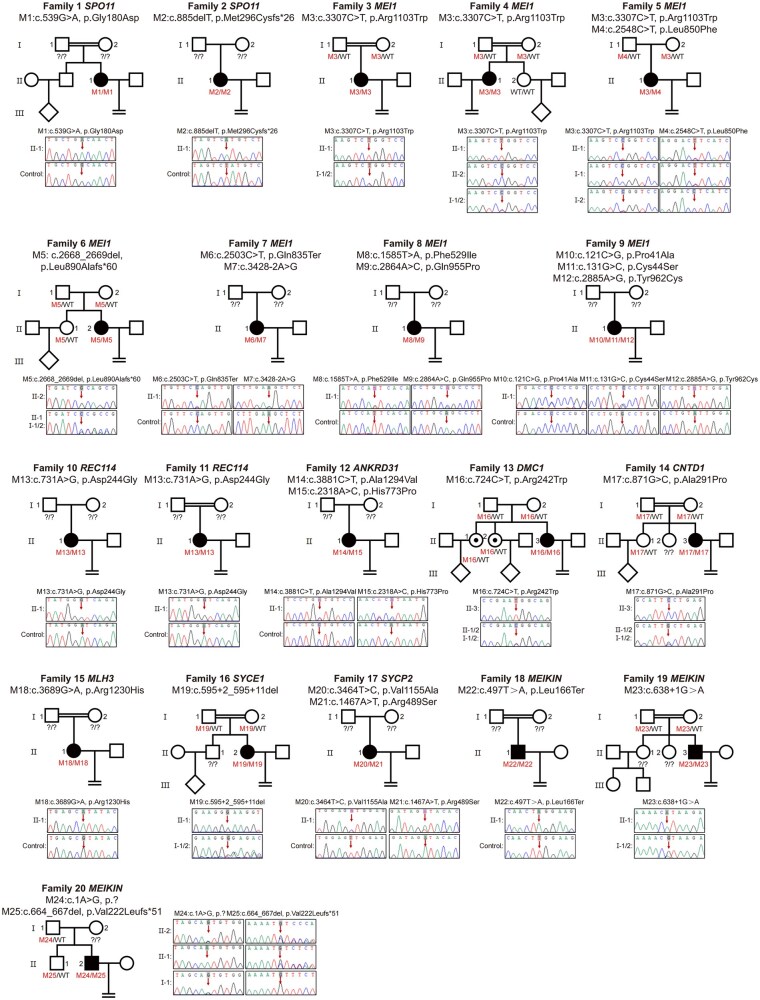
**Pedigrees and Sanger validation of the candidate variants**. Pedigrees of 20 recurrent good-quality cleavage-stage blastulation failure (R-GQBF) families carrying candidate variants in meiotic genes. Filled symbols denote affected individuals. Sanger sequencing chromatograms validating the identified variants are shown adjacent to or below each corresponding pedigree.

Functional enrichment analysis of all candidate genes identified placed meiotic processes among the top five significantly enriched categories (Supplementary Fig. S1). Accordingly, we identified 25 potentially candidate variants across 10 meiotic genes, present in 18.3% (20 of 109) of the study cohort (Fig. 2 and Supplementary Table S2). Among these carriers, 13 patients harbored homozygous variants, while the remaining 7 patients carried two or three heterozygous variants per gene. All variants were validated by Sanger sequencing (Fig. 2). For patients with available family samples, we performed segregation analysis, confirming autosomal recessive inheritance in 15 patients (Fig. 2). Among these carriers, 17 female patients carried 21 distinct variants in 9 genes critical for meiosis prophase I, including synaptonemal complex central element protein 1 (*SYCE1*, GenBank: NM_001143764), synaptonemal complex protein 2 (*SYCP2*, GenBank: NM_014258), meiotic recombination protein SPO11 (*SPO11*, GenBank: NM_012444), meiotic double-stranded break formation protein 1 (*MEI1*, GenBank: NM_152513), meiotic recombination protein REC114 (*REC114*, GenBank: NM_001042367.2), ankyrin repeat domain-containing protein 31 (*ANKRD31*, GenBank: NM_001164443), DNA meiotic recombinase 1 (*DMC1*, GenBank: NM_007068), cyclin N-terminal domain-containing protein 1 (*CNTD1*, GenBank: NM_173478), and mutL homolog 3 (*MLH3*, GenBank: NM_001040108) (Fig. 2 and Table 1). *MEI1* variants were identified in 7 patients, *SPO11* and *REC114* variants were each found in 2 patients, and the other genes were each detected in 1 patient. Three male patients carried 4 distinct variants in meiotic kinetochore protein (*MEIKIN*, GenBank: NM_001303622). Furthermore, we assessed the in‑house control cohort at the individual level and confirmed that no control carried either a homozygous variant or two or more heterozygous variants in the same gene that could potentially form a compound heterozygous genotype.

**Table 1.** Information of pathogenic or likely pathogenic variants in 20 R-GQBF cases.

Family	Location	Gene (transcript ID)	Effect	Amino acids change	rsID	SIFT	Polyphen	MutationTaster	gnomAD	ExAC	ACMG classification
Family 1	chr20:55909834	*SPO11* (NM_012444)	Missense	exon6: c.539G>A, p.Gly180Asp	–	D	D	D	–	–	VUS
Family 2	chr20:55915160		Frameshift deletion	exon11: c.885delT, p.Met296Cysfs*26	–	–	–	–	–	–	VUS
Family 3/4/5	chr22:42180749	*MEI1* (NM_152513)	Missense	exon26: c.3307C>T, p.Arg1103Trp	rs770564395	D	D	P	0	–	VUS
Family 5	chr22:42172109		Missense	exon21: c.2548C>T, p.Leu850Phe	rs200811358	D	D	D	0.0002	0.0002	VUS
Family 6	chr22:42172229-42172230		Frameshift deletion	exon21: c.2668_2669del, p.Leu890Alafs*60	–	–	–	–	–	–	LP
Family 7	chr22:42166924		Stopgain	exon20: c.2503C>T, p.Gln835Ter	–	–	–	D	–	–	LP
	chr22:42190373		Splicing	intron27: c.3428-2A>G	–	–	–	D	–	–	LP
Family 8	chr22:42141935		Missense	exon14: c.1585T>A, p.Phe529Ile	rs535733786	D	D	D	0.0002	0.0001	VUS
	chr22:42177336		Missense	exon23: c.2864A>C, p.Gln955Pro	–	D	D	D	–	–	VUS
Family 9	chr22:42095663		Missense	exon1: c.121C>G, p.Pro41Ala	–	T	D	D	–	–	LB
	chr22:42095673		Missense	exon1: c.131G>C, p.Cys44Ser	–	D	D	D	–	–	VUS
	chr22:42177357		Missense	exon23: c.2885A>G, p.Tyr962Cys	–	D	D	D	–	–	VUS
Family 10/11	chr15:73852187	*REC114* (NM_001042367.2)	Missense	exon6: c.731A>G, p.Asp244Gly	rs1489186376	D	D	D	–	–	VUS
Family 12	chr5:74412494	*ANKRD31* (NM_001164443)	Missense	exon18: c.3881C>T, p.Ala1294Val	rs776341649	D	–	P	–	–	LB
	chr5:74442918		Missense	exon14: c.2318A>C, p.His773Pro	rs550567797	D	–	P	–	–	VUS
Family 13	chr22:38934351	*DMC1* (NM_007068)	Missense	exon11: c.724C>T, p.Arg242Trp	rs1477150457	D	D	D	0	–	VUS
Family 14	chr17:40961431	*CNTD1* (NM_173478)	Missense	exon7: c.871G>C, p.Ala291Pro	rs2054946485	–	B	D	–	–	VUS
Family 15	chr14:75500148	*MLH3* (NM_001040108)	Missense	exon7: c.3689G>A, p.Arg1230His	rs781739661	D	D	D	–	–	VUS
Family 16	chr10:135369474-135369483	*SYCE1* (NM_001143764)	Splicing	intron9: c.595 + 2_595 + 11del	–	–	–	–	0.0001	–	LP
Family 17	chr20:58449002	*SYCP2* (NM_014258)	Missense	exon35: c.3464T>C, p.Val1155Ala	rs6128714	T	B	P	–	–	LB
	chr20:58471521		Missense	exon19: c.1467A>T, p.Arg489Ser	rs769313849	D	D	P	–	–	LB
Family 18	chr5:131257616	*MEIKIN* (NM_001303622)	Stopgain	exon6: c.497T>A, p.Leu166Ter	–	–	–	–	–	–	VUS
Family 19	chr5:131252578		Splicing	intron7: c.638 + 1G>A	rs766888527	–	–	–	–	–	VUS
Family 20	chr5:131281198		Missense	exon1: c.1A>G, p.?	rs534951775	T	D	–	–	–	VUS
	chr5:131247544-131247547		Frameshift deletion	exon8: c.664_667del, p.Val222Leufs*51	rs1159652296	–	–	–	–	–	VUS

rsID, reference SNP cluster ID; D, damaging; B, benign; T, tolerable; P, polymorphism; ACMG, American College of Medical Genetics and Genomics; LP, likely pathogenic; VUS, variant of uncertain significance; LB, likely benign.

*MEI1* appears as a high-risk gene, with 10 variants identified in 7 of the 17 female carriers, including 7 missense variants, and one nonsense, one frameshift, and one intronic variant. This included a recurrent variant p. Arg1103Trp, found in three unrelated patients (Fig. 2 and Table 1). The homozygous variant c.647A>G (p.Asp216Gly) in *REC114* was detected in two patients. Homozygous variants c.724C>T (p.Arg242Trp) in *DMC1* and c.595 + 2_595 + 11del in *SYCE1* were each detected in two patients. In *ANKRD31*, two missense variants c.3881C>T (p.Ala1294Val) and c.2318A>C (p.His773Pro) were detected in one patient. In *SYCP2*, two missense variants c.3464T>C (p.Val1155Ala) and c.1467A>T (p.Arg489Ser) were detected in one patient.

Although all detected variants were novel except the c.1585T>A (p.Phe529Ile) in *MEI1*, the five implicated genes (*REC114*, *DMC1*, *SYCE1*, *ANKRD31*, *SYCP2*) have established roles in female infertility phenotypes (Supplementary Fig. S2). For instance, *MEI1* and *REC114* have been associated with cleavage-stage developmental arrest (Wang *et al.*, 2020; Dong *et al.*, 2021); *MEI1*, *REC114*, and *SYCP2* with recurrent hydatidiform moles (Nguyen *et al.*, 2018); and *ANKRD31*, *DMC1*, and *SYCE1* with primary ovarian insufficiency (POI) (de Vries *et al.*, 2014; He *et al.*, 2018; Wang *et al.*, 2021).Variants in three newly identified female infertility genes included the homozygous missense *SPO11* c.539G>A (p.Gly180Asp) and homozygous frameshift *SPO11* c.885delT (p.Met296Cysfs*26) detected in two patients, respectively, the homozygous missense *CNTD1* c.871G>C (p.Ala291Pro), and the homozygous missense *MLH3* c.3689G>A (p.Arg1230His).

In males, we identified four *MEIKIN* variants in three individuals, two homozygous carriers [nonsense variant c.497T>A (p.Leu166Ter) and splice-site variant c.638 + 1G>A], and one compound heterozygous carrier [promoter variant c.1A>G (p.?) and frameshift variant c.664_667del (p.Val222Leufs*51)] (Fig. 2 and Table 1). *Meikin*, a meiosis-specific kinetochore protein, mediates mono-orientation and centromeric cohesion protection during meiosis I and regulates chromosome alignment in meiosis II (Kim *et al.*, 2015; Maier *et al.*, 2021). *Meikin* knockout causes complete murine infertility (Kim *et al.*, 2015), yet human variants were previously uncharacterized.

### Reproductive and embryonic phenotypes in variant carriers

**Figure 3.**
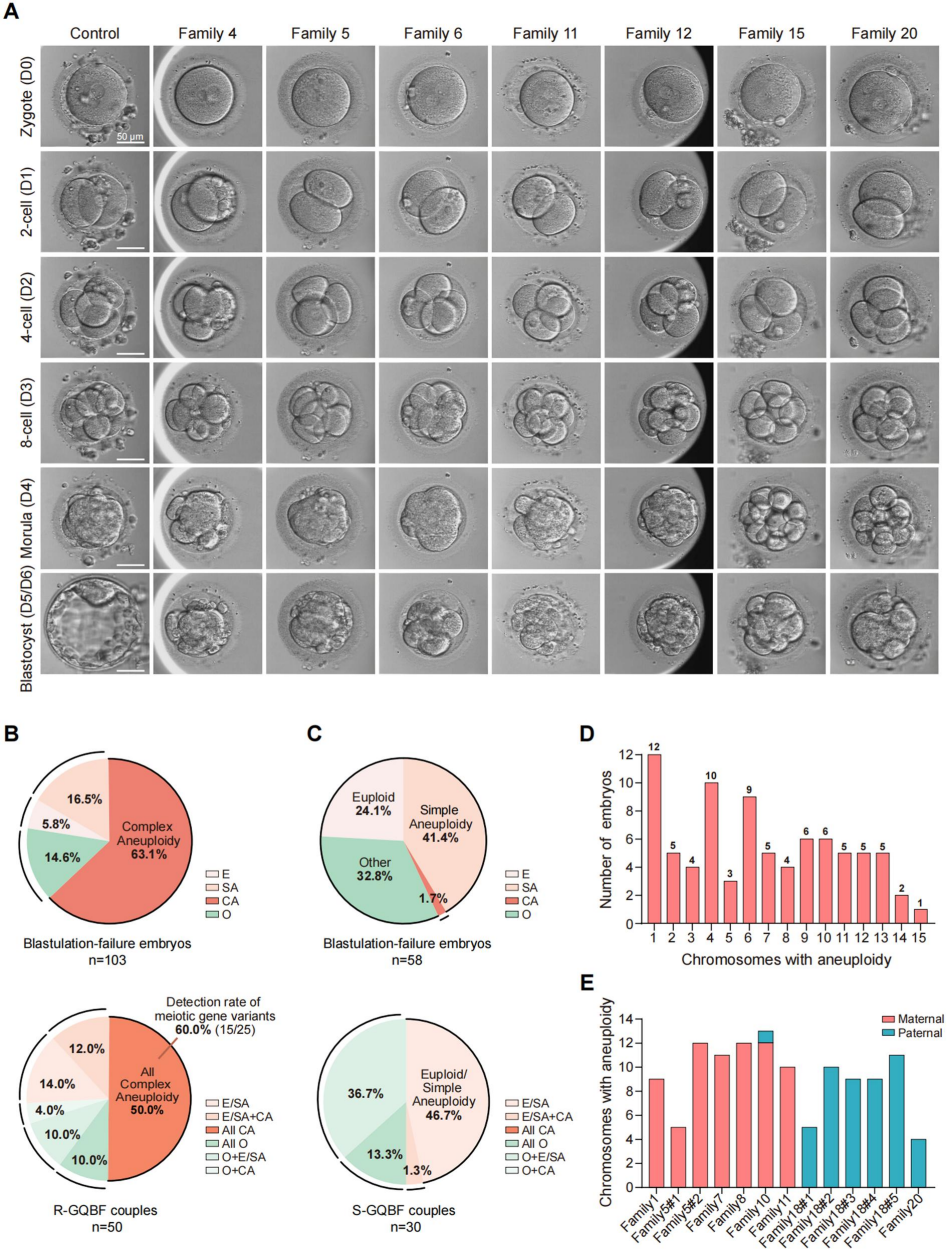
**Embryonic phenotype and genomic profiling of blastulation-failure embryos**. (**A**) Representative embryo morphology from zygote to blastocyst stage in a control who had achieved live birth through ART and in individuals carrying candidate meiotic gene variants. Scale bar, 50 µm. (**B**) Chromosomal classification of 103 blastulation-failure embryos (top) and distribution among 50 recurrent good-quality cleavage-stage blastulation failure (R-GQBF) couples (bottom); all female patients aged ≤38 years. Categories: euploid (E), simple aneuploidy (SA: 1–2 whole-chromosome abnormalities), complex aneuploidy (CA: ≥3 whole-chromosome abnormalities), or other (mosaic or segmental abnormalities). (**C**) Chromosomal classification of 58 blastulation-failure embryos (top) and distribution among 30 sporadic GQBF (S-GQBF) couples (bottom); all female patients aged ≤ 38 years. Categories are the same as in (B). (**D**) Distribution of whole-chromosome aneuploidy in blastulation-failure embryos (82 of 103) from R-GQBF couples. (**E**) Parental origin of aneuploid chromosomes in 13 complex aneuploid blastulation-failure embryos, including 7 embryos from 6 female patients and 6 embryos from 2 male patients. Maternal (red) and paternal (blue) contributions.

We next investigated the clinical characteristics of 20 patients carrying meiotic gene variants. Notably, in all carriers exhibited relatively normal fertilization rates on Day 1 (67.54 ± 17.90%), high cleavage rates on Day 2 (99.55 ± 1.86%), and a substantial proportion of good-quality cleavage-stage embryo on Day 3 (76.57 ± 20.23%). However, these embryos universally showed impaired potential for blastocyst formation, arresting predominantly at the morula stage during extended culture or no pregnancy after routine cleavage-stage embryo transfer according to Chinese expert consensus and routine clinical practice (Fig. 3A and Supplementary Table S3).

Contrary to the established association between meiotic gene variants and POI (Nie *et al.*, 2024), we evaluated ovarian function in meiotic gene variant carriers. We found that 70.6% (12/17) of female carriers exhibited normal ovarian function, as evidenced by regular menstrual cycles, preserved hormonal profiles (FSH: 7.15 ± 2.43 IU/l; LH: 5.14 ± 2.29 IU/l; estradiol: 37.17 ± 14.36 pg/ml), and normal antral follicle counts (AFC: 18.25 ± 5.19) (Supplementary Table S4). Among the remaining female patients, one carrier with a *SYCP2* variant exhibited a PCOS-like phenotype, including oligomenorrhea, elevated AFC (>30), and a reversed FSH/LH ratio (FSH: 1.88 IU/l; LH: 5.51 IU/l). Signs of potential ovarian dysfunction were observed in only four carriers: two with *MEI1* variants, one with a *DMC1* variant, and one with a *CNTD1* variant, who maintained regular cycles and hormonal profiles (FSH: 6.93 ± 1.11 IU/l; LH: 3.72 ± 1.17 IU/l) but demonstrated reduced AFC (5.75 ± 1.26) (Supplementary Table S4).

In contrast to the largely unaffected ovarian function in female carriers, routine semen analysis revealed varying degrees of abnormality in all three male carriers. Significant reductions were observed in sperm concentration, total sperm count, and motility, along with a markedly decreased proportion of normal sperm morphology, exhibiting moderate-to-severe oligoasthenoteratozoospermia (Supplementary Table S5). Further analysis via papanicolaou staining showed sperm with predominantly abnormal head morphology, including large (macrocephalic), small (microcephalic), or absent (acephalic) heads (Supplementary Fig. S3A and B). Toluidine blue staining revealed a decreased proportion of mature sperm with normal chromatin condensation (exhibiting pale blue staining), while the proportion of immature sperm retaining histones (stained dark blue or violet) was significantly increased (Supplementary Fig. S3C and D).

### Predominant complex aneuploidy in blastulation-failure embryos

To further characterize morphologically good cleavage-stage embryos that failed blastulation, we examined the chromosomal constitution of 103 donated blastulation-failure embryos from 50 couples in the R-GQBF cohort using whole-genome sequencing (Fig. 3B and Supplementary Table S6). Embryos were classified as euploid or aneuploid, with aneuploidy further categorized as simple (1–2 whole-chromosome abnormalities), complex (≥3 whole-chromosome abnormalities), or other (mosaic or segmental aneuploidy). We found that complex aneuploidy predominated (63.1%, 65/103) in blastulation-failure embryos, with 50% (25/50) of couples examined producing exclusively complex aneuploid embryos (Fig. 3B). Moreover, 74.4% (61/82) of aneuploid embryos in R-GQBF cases harbored 4–15 chromosomal abnormalities each (Fig. 3D).

To establish a developmentally matched control group, we analyzed 58 blastulation-failure embryos from 30 couples diagnosed with sporadic GQBF (S-GQBF), defined as the isolated failure of a few good-quality cleavage-stage embryos to form blastocysts within a single IVF cycle (female age ≤ 38 years). In this control group, complex aneuploidy was rare, observed in only 1.7% (1/58) of embryos, whereas simple aneuploidy constituted the largest proportion at 41.4% (24/58) (Fig. 3C and Supplementary Table S7). Despite the relatively small sample size of the S‑GQBF control group, the markedly higher prevalence of complex aneuploidy in the R‑GQBF cohort (63.1% vs 1.7%) suggests that complex aneuploidy is a characteristic feature of the recurrent phenotype.

In addition, to provide contextual background on aneuploidy patterns compatible with successful blastocyst development, we analyzed chromosomal data from 15 397 PGT-tested blastocysts obtained from women under 38 years at our center. The overall aneuploidy rate was 20.9% (3214/15 397), among which simple aneuploidy accounted for 96.2% (3091/3214) (Supplementary Fig. S4), indicating that single chromosomal errors are generally compatible with blastocyst formation. Together, these data suggest that severe complex aneuploidy serves as a distinguishing feature of embryos from R-GQBF couples.

Notably, among patients carrying meiotic gene variants, all tested embryos exhibited complex aneuploidy, accounting for 60% (15/25) of R-GQBF couples with exclusively complex aneuploid embryos (Fig. 3B). This indicates the potential value of screening for meiotic gene variants in R-GQBF populations with multiple complex aneuploid embryos.

To further trace the parental origin of chromosomal abnormalities in embryos, we performed CNV origin analysis based on informative SNP patterns among patients carrying meiotic gene variants. We included 13 complex aneuploid embryos for analysis—7 derived from 6 female carriers of variants in *SPO11*, *MEI1*, or *REC114*, and 6 from 2 male carriers of *MEIKIN* variants. Parental origin analysis revealed that chromosomal abnormalities in all 13 embryos predominantly originated from the parent carrying the meiotic gene variants, establishing a direct link between genetic variants and subsequent embryonic chromosomal abnormalities (Fig. 3D and Supplementary Fig. S5).

### Paternal meiotic errors underlie embryonic complex aneuploidy

**Figure 4.**
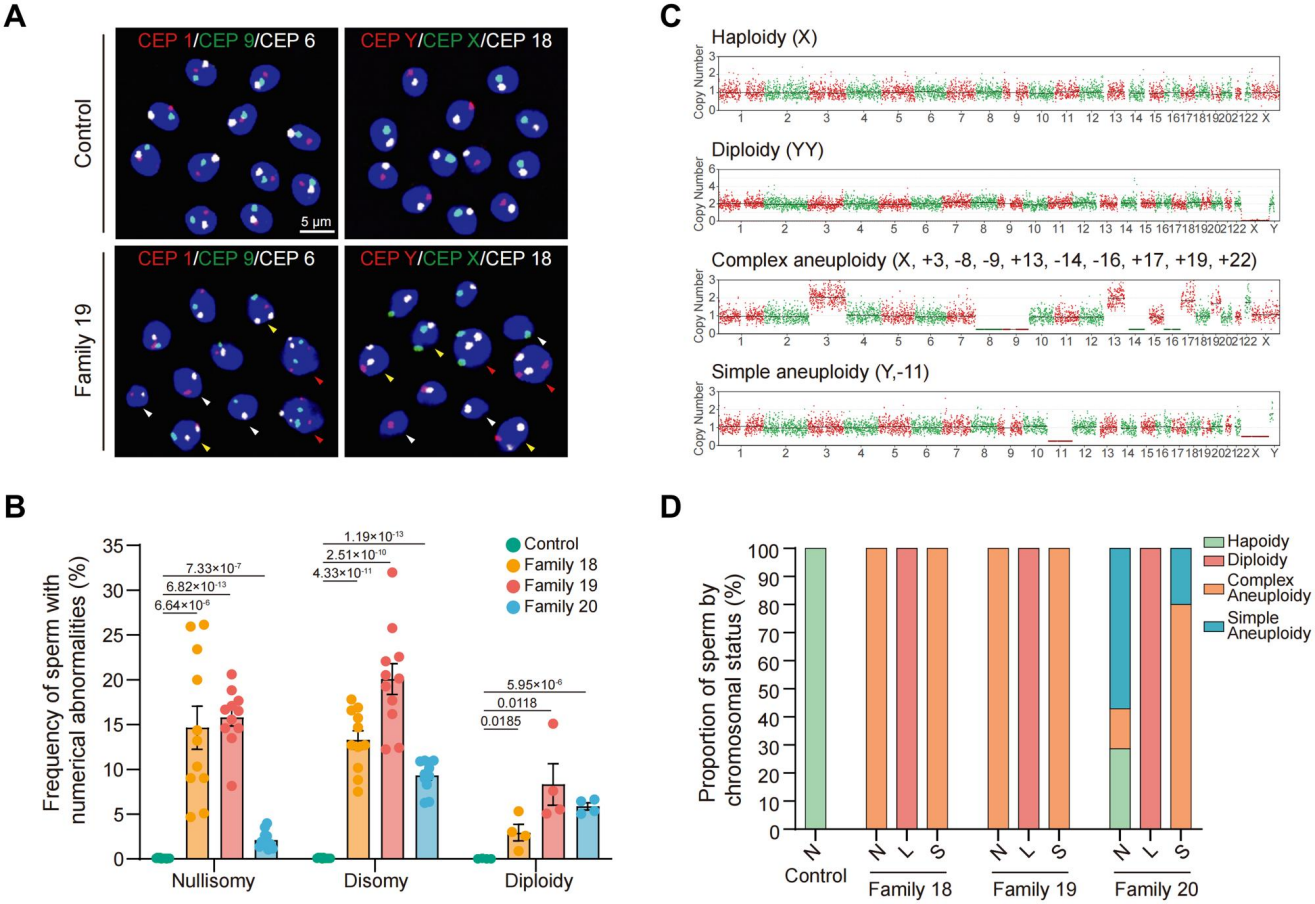
**Chromosomal abnormalities in sperm from individuals carrying *MEIKIN* variants**. (**A**) Fluorescence *in situ* hybridization (FISH) analysis of proven fertile control versus Patient II-1 (Family 19). Chromatin: DAPI (blue). Probe sets: chr1 (red)/chr9 (green)/chr6 (white) and chrY (red)/chrX (green)/chr18 (white). Abnormalities: nullisomy (white arrows), disomy (yellow), diploidy (red). Scale bar, 5 µm. (**B**) Frequencies of disomy and nullisomy (per-chromosome scatter plot) and diploidy (per-probe-set scatter plot) in the control and three patients. Probes: chr1/9/6, 2/12/4, 3/15/7, Y/X/18. Data are presented as mean ± SEM. Exact *P*-values were calculated using two-tailed Student’s *t*-tests. (**C**) Single-sperm whole-genome sequencing examples: haploidy, diploidy, complex aneuploidy (≥3 whole-chromosome abnormalities), and simple aneuploidy (1–2 whole-chromosome abnormalities). (**D**) Distribution of sperm chromosomal status according to head morphology: normal (N), large (L), and small (S).

Since we established that embryonic chromosomal abnormalities originate from parents carrying meiotic gene variants, we next investigated whether these abnormalities stem from meiotic errors in corresponding gametes. Given the scarcity of oocytes, we assessed severe chromosomal abnormalities in sperm from three male patients harboring *MEIKIN* variants. Using sperm FISH analysis covering 12 chromosomes across four assays (≥1000 sperm analyzed per assay), we detected significantly elevated rates of sperm aneuploidy—including disomy, nullisomy, and diploidy—in all three patients carrying *MEIKIN* variants compared to proven fertile controls (Fig. 4A and B and Supplementary Table S8).

To comprehensively evaluate chromosomal integrity, we performed whole-genome sequencing of single sperm. Proven fertile control sperm uniformly exhibited haploidy. In contrast, all normal-headed sperm showed complex aneuploidy from families 18 and 19. However, 28.6% of sperm from family 20 with normal head morphology exhibited haploidy. Macrocephalic sperm displayed extremely high diploidy rates, while microcephalic sperm predominantly exhibited complex aneuploidy (Fig. 4C and D and Supplementary Table S9). Collectively, these results confirm severe chromosomal abnormalities in sperm of *MEIKIN* variant carriers and establish paternal meiotic chromosome segregation errors as the origin of complex aneuploidy observed in corresponding embryos.

### Transcriptomic signatures indicate EGA disruption and stress responses

To explore how complex chromosomal abnormalities lead to blastulation failure in morphologically good cleavage-stage embryos, we performed single-cell RNA sequencing on this type of arrested embryos. After quality control, 15 arrested embryos were retained and integrated with our previously published control embryo data (Leng *et al.*, 2019). Of these, four embryos originated from female variant carriers: one from Family 10 and two from Family 11 (all carrying maternal *REC114* variants), and one from Family 12 (carrying a maternal *ANKRD31* variant). The remaining 11 embryos were derived from the male variant carrier in Family 18, who carried a paternal *MEIKIN* variant.

**Figure 5.**
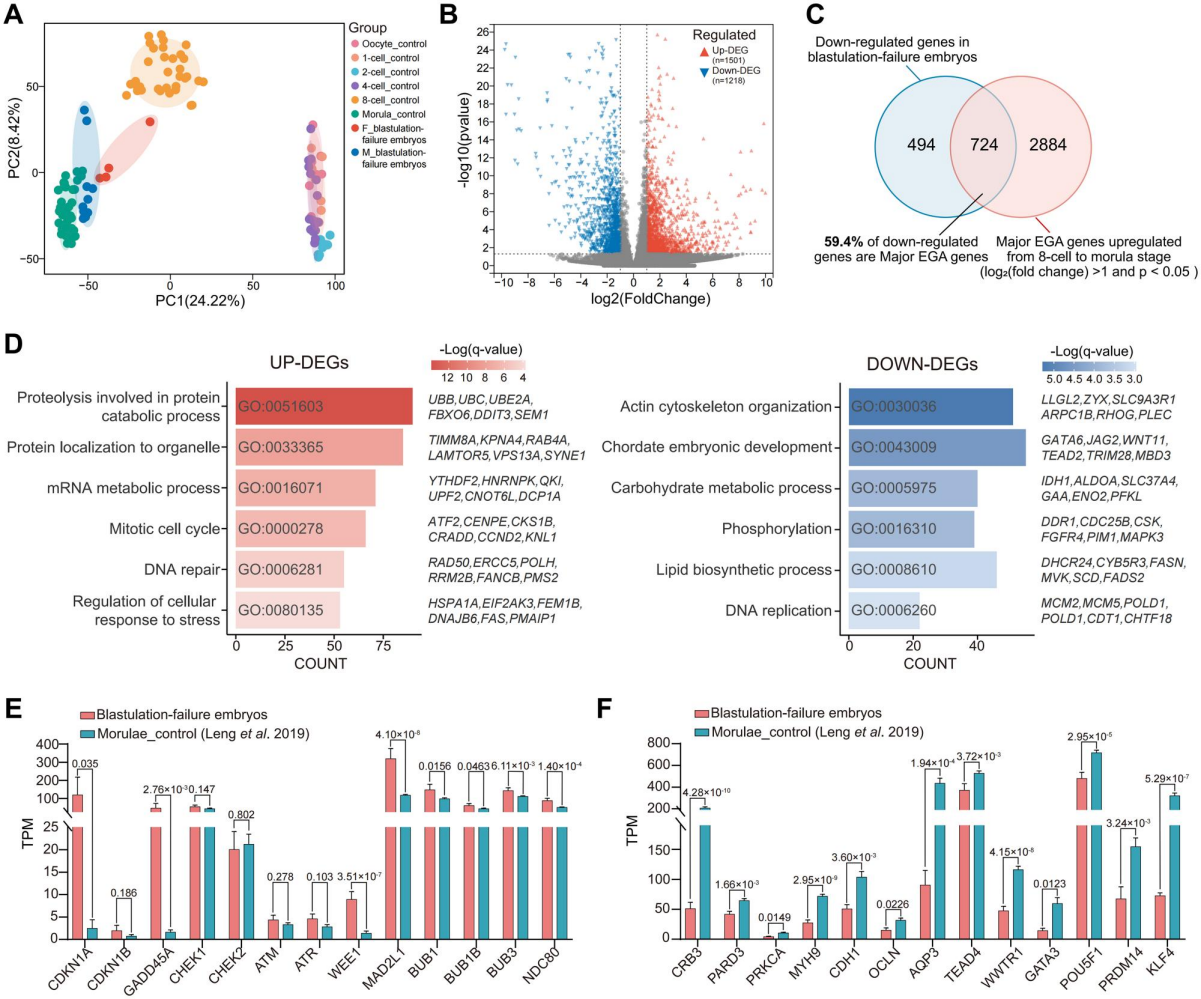
**Transcriptomic profiling of blastulation-failure embryos from individuals with recurrent good-quality cleavage-stage blastulation failure (R-GQBF)**. (**A**) Principal component analysis (PCA) of single-cell transcriptomes from control embryos (publicly available dataset GSE133856 of normally developing embryos) and arrested embryos derived from meiotic variant carriers. Embryos from female carriers are shown in red; those from male *MEIKIN* variant carriers are shown in blue. (**B**) Volcano plot showing differentially expressed genes (DEGs) in blastulation-failure embryos compared to control morula. Upregulated genes are shown in red; downregulated genes in blue. (**C**) Venn diagram showing overlap between down-regulated genes and major embryonic genome activation (EGA) genes. Major EGA genes were defined as those significantly upregulated during from the 8-cell to morula stage [log2 (fold change) ≥ 1 and *P* < 0.05] in dataset GSE133856. (**D**) Gene ontology (GO) enrichment analysis of upregulated and downregulated DEGs. The bar chart depicts the GO terms and representative genes. Color intensity reflects the −log10 (*q*-value) from the enrichment analysis. (**E**) Transcripts per million (TPM) values of representative cell cycle checkpoint regulators. Data are represented as the mean ± SEM. Exact *P*-values were calculated using two-tailed Student’s *t*-tests. (**F**) TPM values of representative genes involved in early lineage specification. Data are represented as the mean ± SEM. Significance as in (E).

Principal component analysis revealed that arrested embryos from both male and female carriers clustered together and both near the morula, suggesting transcriptional arrest at this developmental stage (consistent with morula-stage arrest) (Fig. 5A). Comparative analysis identified 2719 DEGs between blastulation-failure embryos and normal morula, with 1501 upregulated and 1218 downregulated (Fig. 5B). Remarkably, 59.4% of downregulated DEGs were major EGA genes, which are normally upregulated during the 8-cell to morula transition in human embryos, indicating impaired EGA as a characteristic feature of blastulation-failure embryos (Fig. 5C).

Functional enrichment analysis revealed that upregulated genes were primarily associated with proteolysis, mRNA metabolism, mitotic cell cycle, DNA repair, and cellular stress responses (Fig. 5D), consistent with transcriptional patterns frequently observed in aneuploid human cells (Stingele *et al.*, 2012). Given that cell cycle arrest is typically associated with the activation of checkpoint pathways (Panagopoulos and Altmeyer, 2021), we specifically examined the expression patterns of key checkpoint-related genes in our dataset. Several canonical checkpoint regulators, such as *CDKN1A* (MIM: 116899), *GADD45A* (MIM: 126335), *WEE1* (MIM: 193525), and *MAD2L1* (MIM: 601467), were significantly upregulated in blastulation-failure embryos (Fig. 5E). Combined with significant enrichment of DNA repair and stress-response terms, these findings suggest that chromosomal abnormalities may induce DNA damage and activate stress-response pathways, leading to cell cycle checkpoint activation and subsequent developmental arrest.

In contrast, downregulated genes were enriched for essential embryogenic processes, including cytoskeleton organization, embryonic development, carbohydrate metabolism, and DNA replication (Fig. 5D). Since cellular differentiation is critical for blastocyst formation (White *et al.*, 2018), we focused on markers of early lineage specification. We observed suppressed expression of genes governing key blastulation events, including genes critical for polarity establishment [*CRB3* (MIM: 609737), *PARD3* (MIM: 606745), *PRKCA* (MIM: 176960), *MYH9* (MIM: 160775)], compaction [*CDH1* (MIM: 192090), *OCLN* (MIM: 602876)], aquaporin [*AQP3* (MIM: 600170)], trophectoderm determination [*TEAD4* (MIM: 601714), *GATA3* (MIM: 131320)], and pluripotency maintenance [*POU5F1* (MIM: 164177), *PRDM14* (MIM: 611781), *KLF4* (MIM: 602253)] (Fig. 5F). Collectively, these transcriptional patterns are consistent with impaired EGA and disrupted lineage differentiation in arrested embryos, reflecting shared molecular features of blastulation failure rather than variant-specific transcriptional effects.

## Discussion

**Figure 6.**
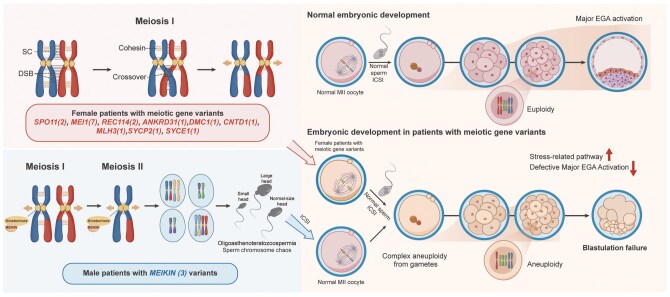
**Proposed schematic model of embryonic arrest in recurrent good-quality cleavage-stage blastulation failure (R-GQBF) patients with meiotic gene variants**. The schematic depicts key meiotic processes: homologous chromosome pairing, recombination involving double-strand breaks (DSBs), meiosis I chromosome segregation, and meiosis II sister chromatid segregation. Pathogenic genes are color-coded (red: female carriers; blue: male carriers), with parentheses indicating variant carrier counts. Maternal or paternal meiotic variants may generate gametes with complex chromosomal abnormalities. Following fertilization with normal gametes, embryos can achieve morphologically normal 8-cell stages despite severe aneuploidy but fail blastulation, likely due to disrupted embryonic genome activation triggered by chromosomal imbalance and aberrant stress pathway activation. Created in BioRender. Xueqin, C. (2026) https://BioRender.com/6lgm4ni.

In summary, our genetic analysis of the R-GQBF cohort revealed candidate variants in 10 meiotic genes across 20 individuals, including 17 female and 3 male patients. Notably, variants in nine meiotic prophase I genes were identified among female carriers, while only three male patients carried variants in *MEIKIN*, a gene functioning both in meiotic I and II. These meiotic defects are associated with severe chromosome segregation errors during gametogenesis, which correlate with the presence of complex aneuploidy in cleavage-stage embryos. Such chromosomal abnormalities coincide with disrupted EGA, downregulation of developmental and lineage-specific gene, and activation of stress-related transcriptional responses, collectively linked to blastulation failure even in morphologically high-quality cleavage-stage embryos (Fig. 6).

While meiotic defects are typically associated with POI (Nie *et al.*, 2024), most female carriers in our study exhibited normal ovarian reserve despite likely producing aneuploid oocytes. This finding suggests that the impact of meiotic gene variants on fertility may be both gene- and variant-specific, with certain variants impairing oocyte chromosome segregation without affecting folliculogenesis. For instance, while N-terminal loss-of-function variants in *ANKRD31* causing complete domain loss led to severe POI (Nie *et al.*, 2024), our study identified only two missense variants in inter-domain linker regions that preserved ovarian reserve. Similarly, although *Spo11* knockout mice exhibit POI (Romanienko and Camerini-Otero, 2000) and complete human loss-of-function would likely recapitulate this phenotype, our patients carried C-terminal non-domain missense and frameshift variants partially preserving protein function, manifesting solely as embryonic developmental defects. These genotype–phenotype correlations warrant future validation through knock-in and knockout mouse functional studies.

Notably, phenotypic heterogeneity related to variant location was observed among the three individuals carrying *MEIKIN* variants. The patient from family 20 presented with milder phenotypes compared to the other two cases, including less severe abnormalities in semen parameters and fewer meiotic errors in sperm. Among morphologically normal-headed sperm, 28.6% were chromosomally normal (Fig. 4D), which may have contributed to the occasional formation of euploid blastocysts with this couple, although the overall blastocyst formation rate remained low. This attenuated phenotype may be attributable to his compound heterozygous variants: a promoter variant and a C-terminus-proximal frameshift mutation (c.664_667del) that likely preserves partial protein function.

Importantly, previously reported gene variants linked to early embryonic arrest have largely been restricted to pre-8-cell stages and often involve maternal-effect genes such as *TLE6* (MIM: 612399), *PADI6* (MIM: 610363), *NLRP2* (MIM: 609364), and *TUBB8* (MIM: 616768) (Xu *et al.*, 2016; Chen *et al.*, 2017; Zheng *et al.*, 2021). In contrast, our study focuses on a distinct phenotype of developmental arrest in morphologically good-quality cleavage-stage embryos occurring after the 8-cell stage, establishing an association between meiotic genes and this phenotype. Additionally, we suggest that this phenotype necessitates considering factors from both parents, as the 8-cell stage represents a critical window for embryonic genome activation where genetic contributions from both parents are essential for subsequent development (Leng *et al.*, 2019). Although most cases involved analysis of only one parent, our findings indicate that embryo arrest may be associated with aneuploidy originating from either parent. This observation, further supported by parental‑origin analysis in a subset of cases (Fig.  3E), highlights the potential relevance of biparental contributions to this developmental phenotype. Our study refines the subclassification of early embryonic developmental phenotypes while extending the phenotypic spectrum of meiotic dysfunction to include later embryonic stages.

Nevertheless, our study has certain limitations. While we confirmed severe chromosomal abnormalities in sperm from males harboring *MEIKIN* variants, we could not directly assess oocytes from female carriers due to limited biological material availability. Although chromosomal origin tracing suggests that complex embryonic aneuploidy likely stem from meiotic chromosome segregation errors in oocytes, future studies employing polar body biopsy may directly observe chromosomal composition in oocytes. Furthermore, while transcriptomic analysis revealed defective EGA and stress pathway activation in blastulation-failure embryos, future multi-omics analyses of oocytes and embryo could further clarify the direct causal relationship between gamete-derived complex aneuploidy and impaired blastocyst formation. Additionally, the disparity in gene discovery rates (9 meiotic genes identified in 17 female patients vs only *MEIKIN* in 3 male patients) may in part reflect cohort bias, as only 16 suspected male-factor cases underwent WES. Expanding the cohort in future work would allow gene‑burden or enrichment analyses to more comprehensively delineate the genetic architecture of this phenotype. Finally, the use of WES primarily captures coding variants and may miss mutations in non‑coding regulatory regions—such as miRNA‑binding sites—that could disrupt maternal transcript clearance and zygotic genome activation. Future studies integrating whole‑genome sequencing and functional assays will be valuable to explore these alternative regulatory pathways in cleavage‑stage arrest.

Collectively, our findings implicate meiotic gene variants as a potential genetic contributor to R-GQBF. These variants are associated with complex, gamete-derived aneuploidy that impair blastulation despite morphologically good cleavage-stage development. From a clinical standpoint, these findings underscore the importance of targeted evaluation in patients who repeatedly fail to generate blastocysts or achieve implantation from good-quality cleavage-stage embryos. In particular, when the blastocyst formation remains below 20% despite a cleavage-stage embryo quality rate exceeding 40%, CNV analysis of blastulation-failure embryos should be considered. In cases of exclusive complex aneuploidy, integration of parental clinical history with genetic screening for meiotic variants and CNV origin analysis may guide more personalized interventions and prevent futile transfer attempts.

## Supplementary Material

hoag039_Supplementary_Data

## Data Availability

The data that support the findings of this study are available from the corresponding author upon reasonable request.
