# Supplementary material for: Meiotic gene variants contribute to recurrent blastulation failure
Source: Hum Reprod Open. 2026 May 7;2026(3):hoag039. doi: 10.1093/hropen/hoag039 (PMC13218795; doi:10.1093/hropen/hoag039)
Supplement: hoag039_Supplementary_Data [file hoag039_supplementary_data.zip › HROPEN-25-0480.R2_-_Supplementary_Materials_Final_EO.pdf]

Supplementary Material

Meiotic Gene Variants Contribute to Recurrent Blastulation Failure

Xueqin Chen, Lizhi Leng, Wenbin He, Weina Li, Lianyue Li, Xing Zhang, Xilin Xu, Jing Dai, Yifan Gu, Pingyuan Xie, Fei Meng, Huiling Hu, Miao Jin, Shujuan Ma, Fei Gong, Guangxiu Lu, Gang Liu, Yueqiu Tan, Ge Lin, Wei Zheng.

Please note that Supplementary Table S1 is provided as a separate Excel file.

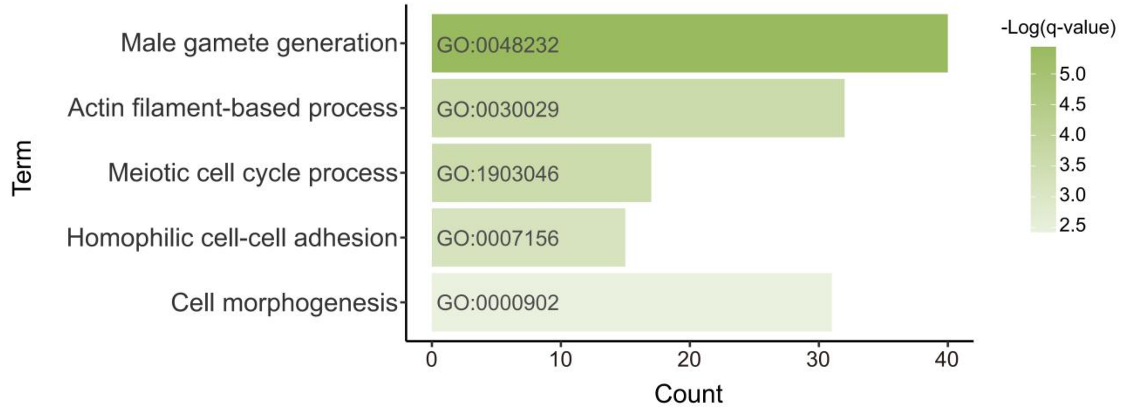

Supplementary Figure S1. Gene Ontology (GO) enrichment analysis of candidate genes with potential biallelic variants. GO enrichment analysis was performed on all candidate genes identified through whole-exome sequencing that harbored potential biallelic variants. The bar chart displays the top five significantly enriched GO terms. The color intensity of each bar reflects the -log10 (q-value) from the enrichment analysis.

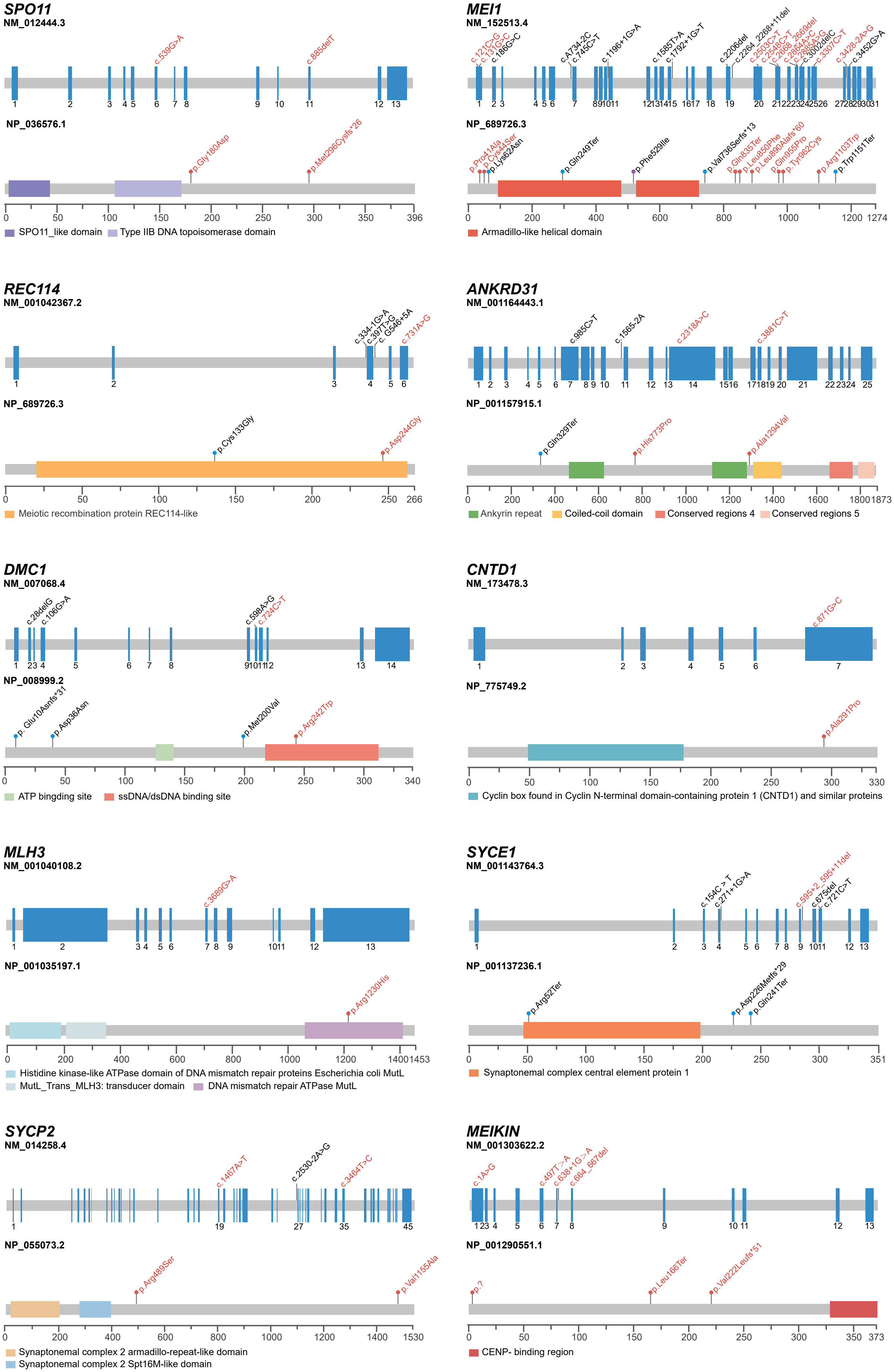

SPO11
NM_012444.3
c.539G>A
c.885delT
1 2 3 4 5 6 7 8 9 10 11 12 13
NP_036576.1
p.Gly180Asp
p.Met296Cysfs*26
0 50 100 150 200 250 300 350 396
SPO11_like domain
Type IIB DNA topoisomerase domain
MEI1
NM_152513.4
c.121G>C
c.131G>C
c.186G>C
c.A734-2C
c.745C>T
c.1196+1G>A
c.1585T>A
c.1792+1G>T
c.2206del
c.2264_2268+11del
c.2503C>T
c.2548C>T
c.2668_2669del
c.2864A>C
c.2885A>G
c.3002delC
c.3307C>T
c.3428-2A>G
c.3452G>A
NP_689726.3
p.Pro41Ala
p.Cys44Ser
p.Lys62Asn
p.Gln249Ter
p.Phe529Ile
p.Val736Serfs*13
p.Gln835Ter
p.Leu850Phe
p.Leu890Alafs*60
p.Gln955Pro
p.Tyr962Cys
p.Arg1103Trp
p.Trp1151Ter
0 200 400 600 800 1000 1200 1274
Armadillo-like helical domain
REC114
NM_001042367.2
c.334-1G>A
c.397T>G
c. G546+5A
c.731A>G
1 2 3 4 5 6
NP_689726.3
p.Cys133Gly
p.Asp244Gly
0 50 100 150 200 250 266
Meiotic recombination protein REC114-like
ANKRD31
NM_001164443.1
c.985C>T
c.1565-2A
c.2318A>C
c.3881C>T
NP_001157915.1
p.Gln329Ter
p.His773Pro
p.Ala1294Val
0 200 400 600 800 1000 1200 1400 1600 1800 1873
Ankyrin repeat
Coiled-coil domain
Conserved regions 4
Conserved regions 5
DMC1
NM_007068.4
c.28delG
c.106G>A
c.598A>G
c.724C>T
NP_008999.2
p. Glu10Asnfs*31
p.Asp36Asn
p.Met200Val
p.Arg242Trp
0 50 100 150 200 250 300 340
ATP bingding site
ssDNA/dsDNA binding site
CNTD1
NM_173478.3
c.871G>C
1 2 3 4 5 6 7
NP_775749.2
p.Ala291Pro
0 50 100 150 200 250 300 330
Cyclin box found in Cyclin N-terminal domain-containing protein 1 (CNTD1) and similar proteins
MLH3
NM_001040108.2
c.3689G>A
1 2 3 4 5 6 7 8 9 10 11 12 13
NP_001035197.1
p.Arg1230His
0 200 400 600 800 1000 1200 1400 1453
Histidine kinase-like ATPase domain of DNA mismatch repair proteins Escherichia coli MutL
MutL_Trans_MLH3: transducer domain
DNA mismatch repair ATPase MutL
SYCE1
NM_001143764.3
c.154C > T
c.271+1G>A
c.595+2_595+11del
c.675del
c.721C>T
1 2 3 4 5 6 7 8 9 10 11 12 13
NP_001137236.1
p.Arg52Ter
p.Asp226Metfs*29
p.Gln241Ter
0 50 100 150 200 250 300 351
Synaptonemal complex central element protein 1
SYCP2
NM_014258.4
c.1467A>T
c.2530-2A>G
c.3464T>C
1 19 27 35 45
NP_055073.2
p.Arg489Ser
p.Val1155Ala
0 200 400 600 800 1000 1200 1400 1530
Synaptonemal complex 2 armadillo-repeat-like domain
Synaptonemal complex 2 Spt16M-like domain
MEIKIN
NM_001303622.2
c.1A>G
c.497T>A
c.638+1G>A
c.664_667del
1 2 3 4 5 6 7 8 9 10 11 12 13
NP_001290551.1
p.?
p.Leu166Ter
p.Val222Leufs*51
0 50 100 150 200 250 300 350 373
CENP- binding region

Supplementary Figure S2. Localization of variants in the genomic and protein structure. Schematic representations of genomic and protein structures showing the locations of identified meiotic gene variants. Novel variants from this study are indicated in red, and previously reported variants are shown in black. All variants, except those in *MEIKIN*, were female-specific, consistent with exclusively female occurrence of reference variants shown.

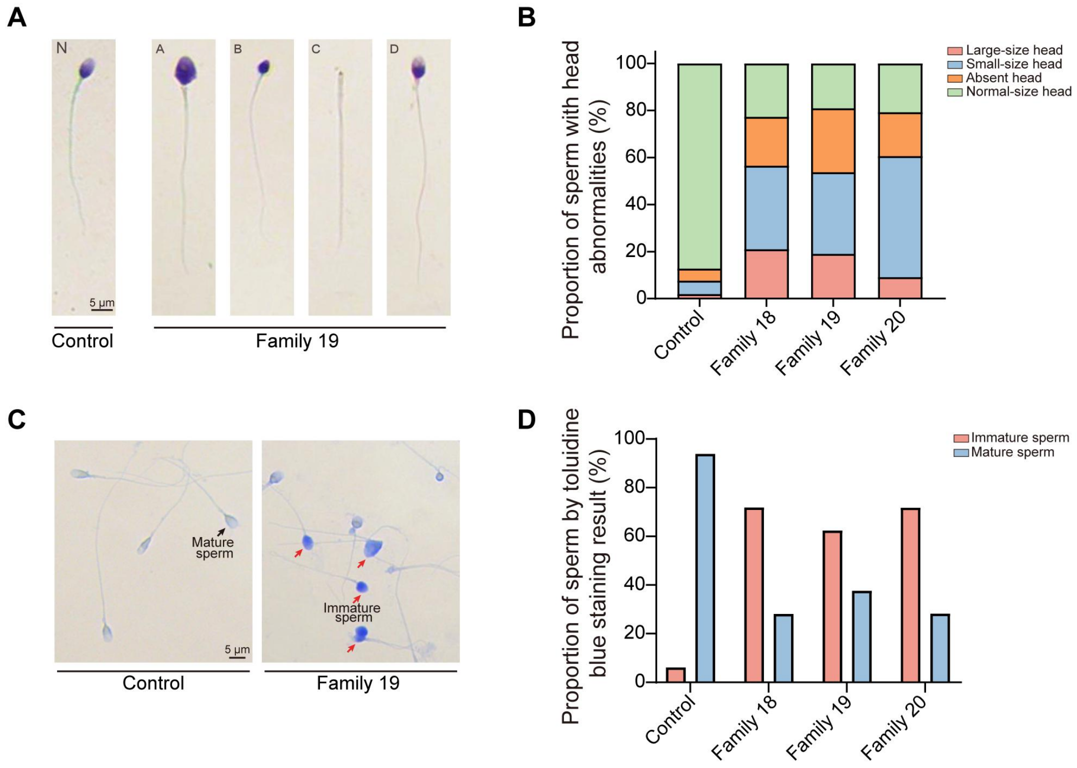

Supplementary Figure S3. Sperm morphology and chromatin integrity in a proven fertile control (free of *MEIKIN* variants) and patients carrying *MEIKIN* variants. (A) Papanicolaou staining of sperm from a control and three patients with *MEIKIN* variants, showing representative head morphology abnormalities. Scale bar = 5 µm. (B) Quantification of distinct categories of sperm head defects in each individual. (C) Toluidine blue staining of sperm from a control and three patients with *MEIKIN* variants. Mature sperm with normal chromatin condensation exhibit pale blue staining (black arrows), while immature sperm retaining histones are stained dark blue or violet (red arrows). Scale bar = 5 µm. (D) Quantification of mature versus immature sperm in the control and the three patients.

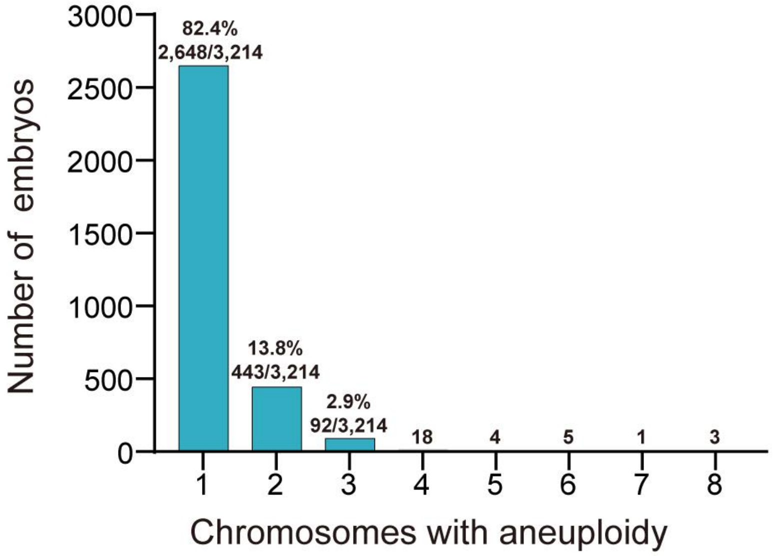

Supplementary Figure S4. Chromosomal aneuploidy distribution in blastocysts. Distribution of whole-chromosome aneuploidy among blastocysts (3,214 of 15,397) from preimplantation genetic testing performed between January 2021 and March 2024 in women aged ≤38 years.

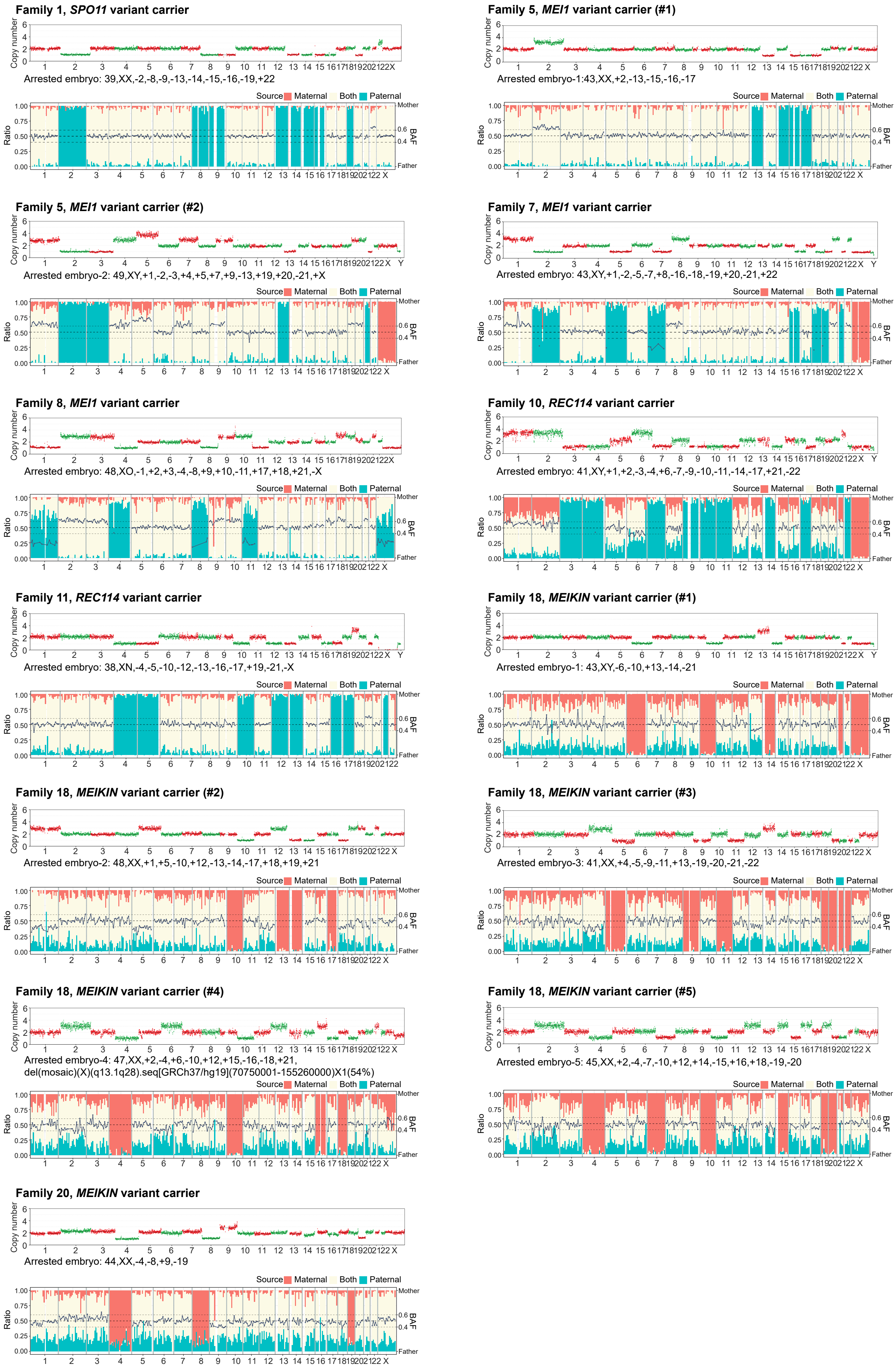

Family 1, SPO11 variant carrier
Arrested embryo: 39,XX,-2,-8,-9,-13,-14,-15,-16,-19,+22
Family 5, MEI1 variant carrier (#1)
Arrested embryo-1:43,XX,+2,-13,-15,-16,-17
Family 5, MEI1 variant carrier (#2)
Arrested embryo-2: 49,XY,+1,-2,-3,+4,+5,+7,+9,-13,+19,+20,-21,+X
Family 7, MEI1 variant carrier
Arrested embryo: 43,XY,+1,-2,-5,-7,+8,-16,-18,-19,+20,-21,+22
Family 8, MEI1 variant carrier
Arrested embryo: 48,XO,-1,+2,+3,-4,-8,+9,+10,-11,+17,+18,+21,-X
Family 10, REC114 variant carrier
Arrested embryo: 41,XY,+1,+2,-3,-4,+6,-7,-9,-10,-11,-14,-17,+21,-22
Family 11, REC114 variant carrier
Arrested embryo: 38,XN,-4,-5,-10,-12,-13,-16,-17,+19,-21,-X
Family 18, MEIKIN variant carrier (#1)
Arrested embryo-1: 43,XY,-6,-10,+13,-14,-21
Family 18, MEIKIN variant carrier (#2)
Arrested embryo-2: 48,XX,+1,+5,-10,+12,-13,-14,-17,+18,+19,+21
Family 18, MEIKIN variant carrier (#3)
Arrested embryo-3: 41,XX,+4,-5,-9,-11,+13,-19,-20,-21,-22
Family 18, MEIKIN variant carrier (#4)
Arrested embryo-4: 47,XX,+2,-4,+6,-10,+12,+15,-16,-18,+21,
del(mosaic)(X)(q13.1q28).seq[GRCh37/hg19](70750001-155260000)X1(54%)
Family 18, MEIKIN variant carrier (#5)
Arrested embryo-5: 45,XX,+2,-4,-7,-10,+12,+14,-15,+16,+18,-19,-20
Family 20, MEIKIN variant carrier
Arrested embryo: 44,XX,-4,-8,+9,-19
Copy number
Ratio
BAF
Source Maternal Both Paternal
Mother
Father

Supplementary Figure S5. Copy-number variant (CNV) origin analysis in blastulation-failure embryos from patients with meiotic gene variants. The upper preimplantation genetic testing for aneuploidy (PGT-A) plot shows chromosomal duplications and deletions in a blastulation-failure embryo. The lower graph corresponds to the PGT-A analysis, where the dashed line represents B allele frequency (BAF) values for single nucleotide polymorphisms (SNPs) in genomic regions where the paternal genotype is AA, the maternal genotype is BB, and the embryonic genotype is AB. BAF values around 0.6 indicate maternal chromosomal duplication, while values near 0.4 suggest paternal duplication. The bar chart represents the ratio of allele combinations (AA, BB, and AB) in the embryo, compared to AABB (paternal AA and maternal BB). The ratios are denoted as AABB-AA-Ratio (blue), AABB-BB-Ratio (red), and AABB-AB-Ratio (light yellow), respectively. Predominance of red bars indicates paternal copy loss, whereas blue bar predominance signifies maternal copy loss.

Supplementary Table S2. Workflow diagram of candidate variant screening.

	Total SNVs	Rare Putatively Functional Variants [a]	Potential Bi-allelic Variant [b]	Variants in Known Meiotic Genes [c]
Family 1	128375	444	67	1 hom (*SPO11*)
Family 2	120649	477	68	1 hom (*SPO11*)
Family 3	106966	480	106	1 hom (*MEI1*)
Family 4	118008	456	54	1 hom (*MEI1*)
Family 5	190613	551	80	2 het (*MEI1*)
Family 6	112512	650	175	1 hom (*MEI1*)
Family 7	127854	472	43	2 het (*MEI1*)
Family 8	119168	476	53	2 het (*MEI1*)
Family 9	119032	459	54	3 het (*MEI1*)
Family 10	127034	428	48	1 hom (*REC114*)
Family 11	108714	507	121	1 hom (*REC114*)
Family 12	123097	441	46	2 het (*ANKRD31*)
Family 13	109813	497	109	1 hom (*DMC1*)
Family 14	109767	489	108	1 hom (*CNTD1*)
Family 15	119993	399	55	1 hom (*MLH3*)
Family 16	178375	551	108	1 hom (*SYCE1*)
Family 17	128249	445	50	2 het (*SYCP2*)
Family 18	126458	499	73	1 hom (*MEIKIN*)
Family 19	127345	434	77	1 hom (*MEIKIN*)
Family 20	126113	433	54	2 het (*MEIKIN*)

Abbreviations: SNV, Single Nucleotide Variant; hom, homozygous; het, heterozygous.

Candidate variants were filtered sequentially as follows: (a) Sequencing quality, allele frequency, and functional impact: sequencing depth >20×; rare variants (minor allele frequency, MAF < 1% in gnomAD and ExAC); and exonic non-synonymous, splice-site, or coding insertions/deletions. (b) Inheritance pattern: biallelic variants in the proband, including homozygous or potential compound heterozygous genotypes (≥2 variants). (c) Gene-based filtering: variants located within a predefined list of 110 meiotic genes (see Supplementary Table S1).

Supplementary Table S3. Embryo characteristics of IVF and ICSI attempts for the affected individuals.

Family	Age	DIF	IVF/ICSI Attempt	Retrieved Oocytes	MII Oocytes	Normal fertilization rate	Embryo cleavage rate	Good-quality embryo rate	Embryo Outcomes
Family 1	32	7	ICSI	12	8	37.5% (3/8)	100% (3/3)	100% (3/3)	1*10-cell and 2*8-cell on Day 3, formed 1*5BA blastocyst on Day 7, trophectoderm chromosomal assessment [48,XN,+7,+7].
			ICSI	12	8	75.0% (6/8)	100% (6/6)	83.33% (5/6)	1*12-cell, 4*8-cell and 1*5-cell, arrested.
Family 2	31	4	ICSI	24	9	55.56% (5/9)	100% (5/5)	60% (3/5)	2*8-cell, 1*6-cell, 1*5-cell and 1*4-cell, arrested
			IVF	16	13	53.85% (7/13)	100% (7/7)	71.43% (5/7)	4*8-cell, 1*6-cell and 2*3-cell on Day 3, formed 1*4BC blastocyst on Day 6, no pregnancy after blastocyst transfer.
Family 3	32	2	IVF	19	18	55.56% (10/18)	100% (10/10)	80% (8/10)	5*8-cell, 2*7-cell, 1*6-cell and 2*4-cell, no pregnancy after 2*8-cell transfer and others arrested.
			ICSI	24	17	76.47% (13/17)	100% (13/13)	76.92% (10/13)	9*8-cell, 1*7-cell, 1*5-cell, 1*4-cell and 1*2-cell, arrested.
Family 4	29	4	IVF	14	14	71.43% (10/14)	100% (10/10)	40% (4/10)	3*8-cell, 1*6-cell, 1*5-cell and 5*4-cell, no pregnancy after 2*8-cell and others arrested.
			IVF	10	10	100% (10/10)	100% (10/10)	50% (5/10)	4*8-cell, 1*6-cell, 2*5-cell, 1*4-cell, 1*3-cell and 1*2-cell, no pregnancy after 2*8-cell and others arrested.
Family 5	30	7	IVF	9	-	-	-	-	no pregnancy after one good cleavage embryo and others arrested.
			IVF	15	-	-	-	-	no pregnancy after two good cleavage embryos and others arrested.
			ICSI	7	4	75.0% (3/4)	100% (3/3)	66.67% (2/3)	1*8-cell, 1*7-cell and 1*4-cell on Day3, formed 1*4BB blastocyst on Day 7, trophectoderm chromosomal assessment [49,XN,+6,+13,+16].
			ICSI	11	8	75.0% (6/8)	100% (6/6)	100% (6/6)	4*8-cell, 1*7-cell and 1*6-cell on Day3, formed 2*5BB blastocyst on Day 6, trophectoderm chromosomal assessment [Triploid,XN,-7,-13,-22]

									and [44,XN,-7,-21].
Family 6	36	11	IVF	12	-	-	-	-	no pregnancy after two good cleavage embryo transfer and others arrested.
			ICSI	13	-	-	-	-	no pregnancy after two good cleavage embryo transfer and others arrested.
			ICSI	4	3	100% (3/3)	100% (3/3)	66.67% (2/3)	2*8-cell and 1*5-cell, arrested.
Family 7	32	8	ICSI	14	7	57.14% (4/7)	100% (4/4)	25% (1/4)	1*8-cell, 2*5-cell and 1*4-cell, arrested.
			ICSI	9	7	71.43% (5/7)	100% (5/5)	80% (4/5)	2*8-cell, 1*7-cell, 1*6-cell and 1*2-cell, arrested.
			IVF	13	10	60.0% (6/10)	100% (6/6)	50% (3/6)	3*8-cell and 1*4-cell, no pregnancy after 3*8-cell and 1*4-cell transfer twice.
			IVF	9	9	44.44% (4/9)	100% (4/4)	100% (4/4)	3*8-cell and 1*6-cell on Day 3, formed 1*4BC blastocyst on Day 6, no pregnancy after blastocyst transfer.
Family 8	34	11	IVF	5	-	-	-	-	no pregnancy after one good cleavage embryo transfer and others arrested.
			IVF	5	-	-	-	-	no pregnancy after one good cleavage embryo transfer and others arrested.
			ICSI	6	5	60.0% (3/5)	100% (3/3)	66.67% (2/3)	2*8-cell and 1*5-cell, arrested.
Family 9	35	14	IVF	6	-	-	-	-	no pregnancy after two good cleavage embryos transfer and others arrested.
			IVF	4	-	-	-	-	no pregnancy after two good cleavage embryos.
			IVF	8	-	-	-	-	no pregnancy after two good cleavage embryos and others arrested.
			ICSI	11	9	33.33% (3/9)	100% (3/3)	100% (3/3)	2*8-cell and 1*6-cell, arrested.
			ICSI	8	5	60.0% (3/5)	100% (3/3)	100% (3/3)	1*9-cell and 2*7-cell, no pregnancy after 1*9-cell and 1*7-cell transfer and others arrested.
Family 10	24	3	Rescue ICSI	18	16	62.50% (10/16)	100% (10/10)	70% (7/10)	4*8-cell, 3*6-cell, 1*5-cell, 1*4-cell and 1*2-cell, arrested.

			IVF	8	8	37.50% (3/8)	100% (3/3)	66.67% (2/3)	1*8-cell, 1*6-cell and 1*4-cell, no pregnancy after 1*8-cell and 1*6-cell transfer.
Family 11	38	7	IVF	20	-	-	-	-	no pregnancy after four good cleavage embryos transfer twice and others arrested.
			ICSI	17	15	80.0% (12/15)	100% (12/12)	91.67% (11/12)	3*12-cell, 5*10-cell, 1*9-cell, 2*8-cell and 1*2-cell arrested.
			IVF+ICSI	12	12	75.0% (9/12)	100% (9/9)	100% (9/9)	2*14-cell, 1*12-cell, 1*10-cell and 5*8-cell, arrested.
Family 12	36	13	IVF	20	-	-	-	-	16 cleavage embryos formed two blastocyst, no pregnancy after two blastocyst transfer.
			IVF	22	-	-	-	-	All of cleavage embryos arrested.
			IVF	18	-	-	-	-	no pregnancy after six good cleavage embryos transfer three times.
			ICSI	16	14	92.86% (13/14)	92.31% (12/13)	75.0% (9/12)	4*8-cell, 4*7-cell, 1*6-cell, 1*5-cell, 1*4-cell and 1*2-cell, arrested.
			ICSI (Husband' s sperm and donor sperm)	15	13	61.54% (8/13)	100% (8/8)	100% (8/8)	1*12-cell, 1*11-cell, 1*10-cell, 4*8-cell and 1*7-cell, arrested.
Family 13	35	11	IVF	2	1	-	-	-	no pregnancy after 1*8-cell transfer.
			IVF	4	4	-	-	-	All of cleavage embryos arrested.
			IVF	6	6	-	-	-	no pregnancy after 2*8-cell and others arrested.
			ICSI	8	6	-	-	-	no pregnancy after 2*8-cell and others arrested.
			IVF	6	5	-	-	-	no pregnancy after 2*8-cell and others arrested.
Family 14	38	11	IVF	8	-	-	-	-	no pregnancy after two good cleavage embryos transfer.
			IVF	5	-	-	-	-	no pregnancy after three good cleavage embryos transfer twice.
			IVF	4	-	-	-	-	no pregnancy after two good cleavage embryos transfer.
			IVF	8	-	-	-	-	no pregnancy after two good cleavage embryos transfer.

			IVF	2	-	-	-	-	no pregnancy after one good cleavage embryos transfer.
			ICSI	2	2	50% (1/2)	100% (1/1)	100% (1/1)	1*12-cell, arrested.
Family 15	33	8	IVF	7	5	60% (3/5)	100% (3/3)	66.67% (2/3)	1*10-cell, 1*7-cell and 1*4-cell, arrested.
			IVF	13	13	46.15% (6/13)	100% (6/6)	100% (6/6)	3*8-cell and 3*7-cell, arrested.
Family 16	35	5	IVF	14	-	-	-	-	no pregnancy after 2*9-cell and 2*8-cell transfer twice and others arrested.
			ICSI	8	6	100% (6/6)	100% (6/6)	66.67% (4/6)	3*8-cell, 1*6-cell, 1*5-cell and 1*4-cell, arrested.
Family 17	35	5	IVF+ICSI	15	13	69.23% (9/13)	100% (9/9)	88.89% (8/9)	1*16-cell, 6*8-cell, 1*7-cell and 1*5-cell, no pregnancy after 2*8-cell transfer and others arrested.
			ICSI	14	10	80% (8/10)	100% (8/8)	100% (8/8)	1*10-cell, 5*8-cell, and 2*6-cell, no pregnancy after 2*8-cell transfer and others arrested.
Family 18	31	3	ICSI	14	13	92.31% (12/13)	91.66% (11/12)	45.45% (5/11)	1*10-cell, 1*8-cell, 1*7-cell, 2*6-cell, 2*5-cell, 1*4-cell, 1*3-cell and 2*2-cell, arrested.
			Husband's sperm ICSI	10	8	75.0% (6/8)	100.0% (6/6)	66.67% (4/6)	1*10-cell, 2*8-cell, 1*6-cell, 1*5-cell and 1*3-cell, arrested.
			Donor sperm IVF	10	10	100.0% (10/10)	100.0% (10/10)	70.0% (7/10)	3*8-cell, 3*7-cell, 1*6-cell, 1*5-cell, 1*4-cell and 1*3-cell on Day 3, formed 4*5BC and 3*4BC blastocyst on Day 6, pregnancy after one blastocyst transfer.
Family 19	33	5	ICSI	12	8	87.50% (7/8)	100.0% (7/7)	57.14% (4/7)	1*10-cell, 1*8-cell, 2*6-cell, 1*5-cell, 1*4-cell and 1*2-cell, no pregnancy after 1*10-cell and 1*8-cell transfer and others arrested.
			ICSI	14	12	83.33% (10/12)	100% (10/10)	70.0% (7/10)	4*8-cell, 2*7-cell, 1*6-cell, 1*5-cell, and 2*4-cell, no pregnancy after 2*8-cell transfer and others arrested.
			Donor sperm ICSI	12	7	85.71% (6/7)	66.66% (4/6)	75.0% (3/4)	2*8-cell, 1*6-cell and 1*2-cell on Day 3, formed 2*5BC blastocyst on Day 6 and 1*6BC blastocyst on Day 7, pregnancy after one blastocyst transfer.

			ICSI	18	-	-	-	-	no pregnancy after two good cleavage embryos transfer and others arrested.
			ICSI	18	-	-	-	-	no pregnancy after two good cleavage embryos transfer.
Family 20	32	3	ICSI	14	12	66.67% (8/12)	100% (8/8)	75.0% (6/8)	4*8-cell, 2*6-cell, 2*4-cell on Day 3, formed 1*4BC blastocyst on Day 7, trophectoderm chromosomal assessment [45,XN,+9,-16,-21].
			Husband's sperm ICSI	4	4	50.00% (2/4)	100% (2/2)	100% (2/2)	2*8-cell on Day 3, formed 1*6AB blastocyst on Day 6, trophectoderm chromosomal assessment [46,XN].
			Donor sperm ICSI	3	3	66.67% (2/3)	100% (2/2)	100% (2/2)	1*9-cell,1*8-cell and 1*2-cell on Day 3, formed 1*4BB and 1*5BB blastocyst on Day 6.

Supplementary Table S4. Clinical characteristics of the female patients.

Characteristic	Group	Maternal age (years)	Maternal BMI (kg/m2)	Basal FSH (mIU/ml)	Basal LH (mIU/ml)	Basal E2 (pg/ml)	AMH (ng/ml)	AFC
Family 1	Normal Ovarian Function	32	21.25	7.03	4.66	49.2	2.503	16
Family 2		31	23.81	8.41	6.72	27.7	-	19
Family 3		32	20.28	5.94	5.27	54.5	2.66	24
Family 4		29	23.73	7.15	5.63	22	1.828	16
Family 5		30	20.03	6.5	2.79	34.2	-	17
Family 7		32	20.32	6.62	3.33	32.83	1.617	12
Family 9		35	21.10	7.6	4.56	25	1.08	11
Family 10		24	21.48	0.9	0.63	12.42	-	25
Family 11		38	24.61	8.71	8.38	48.4	5.9	22
Family 12		36	22.31	6.93	7.71	52	-	26
Family 15		33	19.98	11.2	7.8	55.70	2.05	19
Family 16		35	24.91	8.88	4.22	32.2	1.532	12
Mean±SD		31.88±4.08	21.98±1.83	7.15±2.43	5.14±2.29	37.17±14.36	2.40±1.51	18.25±5.19
Family 6	Potential Ovarian Dysfunction	36	22.03	5.78	3.08	43.5	1.16	6
Family 8		34	18.67	8.4	4.55	31.7	0.61	4
Family 13		35	22.22	7.06	2.40	47.7	1.69	7
Family 14		38	19.20	6.46	4.85	48.8	-	6
Mean±SD		35.75±1.71	20.53±1.86	6.93±1.11	3.72±1.17	42.93±7.82	1.15±0.54	5.75±1.26
Family 17	PCOS-like	35	23.19	1.88	5.51	27.39	24.347	>30
Reference			18.5-24.0	3.5-12.5	2.4-12.6	20-80	0.88-11.53	10-20

Data are expressed as the mean ± standard deviation. AMH data were missing for 5 female patients.

Abbreviations: E2, estrogen; AMH, anti-Mullerian hormone; AFC, antral follicle count.

Supplementary Table S5. Semen parameters in individuals carrying *MEIKIN* variants.

Semen Parameters	Family 18	Family 19	Family 20	Reference
Semen volume (ml)	3.50	2.93	4.00	>1.50
Sperm concentration (10^6/ml)	2.15	6.12	8.48	>15.00
Total sperm counts (TSC)	7.52	17.93	33.92	>39.00
Total motile spermatozoa [(PR+NP) %]	12.00	38.40	17.31	>40.00
Progressive motility (PR%)	8.00	28.00	9.62	>32.00
Rate of normal morphology spermatozoa (%)	0.97	0.99	1.12	>4.00

Supplementary Table S6. Chromosome copy number results for blastulation-failure embryos in the R-GQBF cohort.

Patients	Family	No. of embryo	Single-embryo-based whole-genome sequencing	Classified
P1	Family 1	#1	39,XX,-2,-8,-9,-13,-14,-15,-16,-19,+22	Complex aneuploidy
		#2	41,XY,+1,-2,+3,-5,-6,+8,+11,-12,-13,-14,-17,-18,-19,-20,+X	Complex aneuploidy
		#3	51,XO,+4,+6,+8,+10,+11,+12,-13,-14,+18,+20,-X	Complex aneuploidy
P2	Family 2	#1	47,XY,+(mosaic)(2)(69%),+7,-8,+14,-17,+19,-(mosaic)(21)(66%),-(mosaic)(X)(37%)	Complex aneuploidy
		#2	46,XX,dup(mosaic)(1)(p36.33-q31.1)(151.8Mb)(63%),+3,-(mosaic)(4)(52%),+(mosaic)(6)(36%),-(mosaic)(8)(64%),-(mosaic)(13)(62%),+(mosaic)(14)(62%),-16,-(mosaic)(17)(69%),-(mosaic)(18)(65%),-19,+21	Complex aneuploidy
P3	Family 4	#1	51,XY,+3,+4,+6,-7,+8,+14,dup(mosaic)(18)(p11.22-q23)(69.02Mb)(63%),+20	Complex aneuploidy
P4	Family 5	#1	43,XX,+2,-13,-15,-16,-17	Complex aneuploidy
		#2	49,XY,+1,-2,-3,+4,+5,+7,+9,-13,+19,+20,-21,+X	Complex aneuploidy
P5	Family 6	#1	47,XO,+2,+3,+5,-7,+11,+16,-18,-22,-X	Complex aneuploidy
		#2	43,XO,-3,+4,-5,-7,-10,-11,-14,+15,+16,+(mosaic)(19)(32%),+22,-X	Complex aneuploidy
P6	Family 7	#1	43,XY,+1,-2,-5,-7,+8,-16,-18,-19,+20,-21,+22	Complex aneuploidy
		#2	49,XY,+1,+3,+5,del(6)(p25.3-q16.1)(93.76Mb),del(6)(q16.1-q23.2)(36.84Mb),+7,-10,+13,+14,-16,-18,-19,+20,+22,-X	Complex aneuploidy
P7	Family 8	#1	44,XX,-4,-6,-7,-9,-10,+11,+12,-13,+14,+21	Complex aneuploidy
		#2	48,XO,-1,+2,+3,-4,-8,+9,+10,-11,+17,+18,+21,-X	Complex aneuploidy
		#3	46,XX,-2,+5,+8,+9,+10,+14,-15,-16,+17,-19,-20,-21	Complex aneuploidy
P8	Family 10	#1	41,XY,+1,+2,-3,-4,+6,-7,-9,-10,-11,-14,-17,+21,-22	Complex aneuploidy

P9	Family 11	#1	38,XN,-4,-5,-10,-12,-13,-16,-17,+19,-21,-X	Complex aneuploidy
		#2	47,XY,+1,+2,+4,dup(mosaic)(5)(q11.2-q35.3)(50700001-180740000)X3(45.89%) ,-8,-11,+12,-13,-15,-16,-17,+18,+20,+22	Complex aneuploidy
		#3	38,XN,-2,-5,-8,+9,+10,-11,-12,-14,-15,-16,-18,+21,-22,-X	Complex aneuploidy
P10	Family 14	#1	44,XX,-2,-3,-6,-8,+9,-10,+11,-12,+(mosaic)(13)(33%),-14,+15,+21,+22	Complex aneuploidy
		#2	46,XX,-(mosaic)(1)(63%),-2,+13,-(mosaic)(14)(62%),-(mosaic)(15)(57%),+18,-(mosaic)(21)(61%),-22	Complex aneuploidy
P11	Family 15	#1	48,XO,dup(mosaic)(4)(q22.3-q31.3)(55.8Mb)(33%),del(mosaic)(4)(q31.3-q35.2)(34.4Mb)(30%),+9,+15,+16,+18,-21,-X	Complex aneuploidy
		#2	50,XX,+4,+7,+9,+10,+14,-15,-20,+22	Complex aneuploidy
P12	Family 16	#1	42,XX,-(mosaic)(4)(55%),del(mosaic)(5)(p15.33q12.1)(51.8Mb)(58%),-(mosaic)(7)(53%),-9,-(mosaic)(11)(65%),-13,-14,-17,-(mosaic)(20)(64%)	Complex aneuploidy
		#2	44,XY,+1,-3,-4,+5,-6,-9,-11,+15,-16,+17,-19,+20,+21,-22	Complex aneuploidy
		#3	45,XY,-1, del(2)(q11.2q37.3)(142.8Mb),+(mosaic)(3)(56%),-8,-9,+11,-16,+17,-(mosaic)(19)(68%),+22	Complex aneuploidy
P13	Family 18	#1	43,XY-6,-10,+13,-14,-21	Complex aneuploidy
		#2	48,XX,+1,+5,-10,+12,-13,-14,-17,+18,+19,+21	Complex aneuploidy
		#3	41,XX,+4,-5,-9,-11,+13,-19,-20,-21,-22	Complex aneuploidy
		#4	47,XX,+2,-4,+6,-10,+12,+15,-16,-18,+21,del(mosaic)(X)(q13.1q28).seq[GRCh37/hg19](70750001-155260000)X1(54%)	Complex aneuploidy
		#5	45,XX,+2,-4,-7,-10,+12,+14,-15,+16,+18,-19,-20	Complex aneuploidy

P14	Family 19	#1	42,XY,+7,-8,-13,+14,-15,-18,-20,-22	Complex aneuploidy
P15	Family 20	#1	44,XX,-4,-8,+9,-19	Complex aneuploidy
		#1	44,XX,+1,-2,-7,-22	Complex aneuploidy
P16	-	#2	46,XY,-3,-4,+11,+X	Complex aneuploidy
		#3	46,XX,+1,-3,-11,+12	Complex aneuploidy
P17	-	#1	45,XY,-19	Simple aneuploidy
		#2	47,XY,+19	Simple aneuploidy
P18	-	#1	46,XX,del(mosaic)(1)(p12-q44)(128.52Mb)(40%)	Other
P19	-	#1	48,XY,-(mosaic)(1)(56%),+3,-(mosaic)(4)(33%),-5,-(mosaic)(7)(43%),-(mosaic)(8)(49%),+(mosaic)(10)(44%),+(mosaic)(12)(62%),-(mosaic)(13)(45%),+14,-(mosaic)(16)(52%),+17,+18,-21,+(mosaic)(X)(47%)	Complex aneuploidy
		#2	48,XX,+15,+22	Simple aneuploidy
P20	-	#1	46,XY	Euploidy
		#2	45,XX,dup(mosaic)(5)(p15.33-p15.2)(10.4Mb)(54%),del(7)(p22.3-p12.1)(52.4Mb),del(7)(p12.1-q36.3)(92.4Mb)	Other
P21	-	#1	43,XY,-1,-3,-4,+6,+9,-11,+13,+15,-16,+(mosaic)(17)(34%),-19,-20,+22,-X, -(mosaic)(Y)(33%)	Complex aneuploidy
		#2	42,XO,+(mosaic)(6)(52%),-10,+11,-12,-16,-19,-X	Complex aneuploidy
P22	-	#1	46,XY,dup(2)(q13-q37.3)(131.7Mb)	Other
P23	-	#1	46,XX	Euploidy
P24	-	#1	46,XY,del(mosaic)(18)(q12.2-q23)(40.0Mb)(66%)	Other

		#2	46,XX,dup(4)(q32.1-q32.2)(6.0Mb),dup(18)(q22.1-q23)(9.8Mb)	Other
P25	-	#1	48,XY,+1,+3,-(mosaic)(4)(65%),-5,-6,-7,-(mosaic)(9)(42%),+(mosaic)(10)(63%),+(mosaic)(12)(62%),<br>-(mosaic)(13)(32%),-(mosaic)(14)(36%),+(mosaic)(15)(44%),+16,-(mosaic)(17)(42%),+21,+X	Complex aneuploidy
		#2	44,XY,-2,+4,dup(6)(p25.3-p11.2)(57.2Mb),-(mosaic)(9)(39%),+13,-14,-15,-(mosaic)(17)(66%),-18	Complex aneuploidy
P26	-	#1	46,XX,del(mosaic)(9)(q32-q34.3)(22.6Mb)(31%),dup(mosaic)(15)(q13.1-q26.3)(70.0Mb)(59%)	Other
		#2	46,XY,-(mosaic)(5)(59%),-(mosaic)(6)(32%),-(mosaic)(9)(44%),-(mosaic)(12)(37%),-(mosaic)(14)(37%),-(mosaic)(16)(32%),-(mosaic)(17)(56%),-(mosaic)(18)(33%),-(mosaic)(19)(44%),-(mosaic)(22)(43%)	Other
P27	-	#1	47,XY,-(mosaic)(6)(42%),+16,+(mosaic)(X)(36%),-(mosaic)(Y)(34%)	Simple aneuploidy
		#2	49,XY,+(mosaic)(1)(68%),-(mosaic)(8)(47%),+9,+10,+X	Complex aneuploidy
P28	-	#1	48,XY,+11,+X,-(mosaic)(Y)(65%)	Simple aneuploidy
		#2	46,XY,-(mosaic)(1)(54%),-(mosaic)(2)(57%),-(mosaic)(4)(49%),-(mosaic)(9)(55%),+(mosaic)(13)(60%),+(mosaic)(X)(63%)	Other
P29	-	#1	45,XY,-9	Simple aneuploidy
		#2	45,XX,+1,+2,-5,-10,-11,+13,+18,-19,-21	Complex aneuploidy
		#3	42,XX,-1,-3,-6,-13	Complex aneuploidy
		#4	44,XY,-3,-4,+6,-10,-17,+21,+(mosaic)(22)(64%),<br>+(mosaic)(X)(36%)	Complex aneuploidy
P30	-	#1	46,XX,+(mosaic)(13)(39%),dup(mosaic)(14)(q11.2-q21.1)(20.42Mb)(36%)	Other
		#2	47,XY,-(mosaic)(1)(43%),-(mosaic)(5)(34%),+(mosaic)(15)(31%),-(mosaic)(18)(59%),+20	Simple aneuploidy
P31	-	#1	46,XX,-(mosaic)(2)(35%),+(mosaic)(3)(69%),+(mosaic)(6)(53%),+(mosaic)(13)(50%),-(mosaic)(14	Other

			)(44%),+(mosaic)(15)(50%),-(mosaic)(18)(30%),+(mosaic)(20)(36%)	
		#2	46,XX,+(mosaic)(2)(43%),-(mosaic)(8)(30%),-(mosaic)(10)(34%),-(mosaic)(12)(34%),+(mosaic)(13)(45%),+(mosaic)(14)(51%),-(mosaic)(18)(40%),-(mosaic)(19)(48%),+(mosaic)(22)(50%),+(mosaic)(X)(32%)	Other
P32	-	#1	45,XY,+(mosaic)(8)(41%),del(mosaic)(9)(p13.3-q34.3)(106.28Mb)(38%),dup(mosaic)(18)(q21.33-q23)(17.0Mb)(34%),-20,+(mosaic)(21)(41%)	Simple aneuploidy
		#2	45,XY,+(mosaic)(2)(43%),-12,+(mosaic)(13)(50%),+(mosaic)(16)(57%),-(mosaic)(20)(64%),del(mosaic)(22)(q11.21-q13.32)(30.74Mb)(65%),-(mosaic)(Y)(35%)	Simple aneuploidy
P33	-	#1	42,XY,-4,-6,-8,+13,-15,-21	Complex aneuploidy
		#2	48,XO,+1,+2,+4,-(mosaic)(5)(44%),-10,+14,-(mosaic)(15)(52%),-(mosaic)(18)(32%),-X	Complex aneuploidy
P34	-	#1	41,XY,-2,-5,-6,+8,-9,-10,-12,-18,+21,-22,+X	Complex aneuploidy
		#2	45,XY,-4,-5,+(mosaic)(8)(30%),-10,-13,+16,+17,+21	Complex aneuploidy
P35	-	#1	42,XY,+1,-2,-6,-11,+12,-15,-16,-18,-20,+22	Complex aneuploidy
		#2	46,XY,-1,+2,-5,+(mosaic)(6)(64%),-7,+12,+14,-17,+18,-19,+21,-(mosaic)(Y)(31%)	Complex aneuploidy
		#3	46,XY,+2,+4,+5,-8,-9,-10,-11,del(mosaic)(12)(q12-q24.33)(89.0Mb)(41%),-13,+15,-16,+21,+22	Complex aneuploidy
P36	-	#1	45,XX,-15	Simple aneuploidy
		#2	45,XY,+(mosaic)(3)(35%),-8	Simple aneuploidy
P37	-	#1	46,XY	Euploidy
P38	-	#1	42,XY,-4,-6,-10,-12	Complex aneuploidy
		#2	46,XX,-(mosaic)(5)(51%),del(mosaic)(7)(34%)	Other
P39	-	#1	44,XX,-2,-10,del(8)(p23.3-p22)(10.2Mb)	Simple aneuploidy
P40	-	#1	45,Y,+2,-5,+7,-12,-21 ,-X	Complex aneuploidy

		#2	43,OY,-22,-X	Simple aneuploidy
P41	-	#1	48,XX,-1,+2,-4,+5,+8,+11,-12,-14,-17,+19,+20,+22	Complex aneuploidy
		#2	43,OY,-1,+4,-5,+6,-8,-11,-12,+14,-16,-17,+18,+20,-X	Complex aneuploidy
		#3	48,XY,+(mosaic)(2)(69%),+(mosaic)(3)(67%),+(mosaic)(6)(63%),-9,+(mosaic)(10)(65%),+(mosaic)(13)(66%),+14,+(mosaic)(15)(67%),+16,+17,-20,+21	Complex aneuploidy
P42	-	#1	48,XY,+2,-(mosaic)(4)(44%),-(mosaic)(6)(50%),-(mosaic)(7)(48%),-(mosaic)(9)(46%),-(mosaic)(10)(45%),+15,-(mosaic)(17)(55%),-(mosaic)(18)(44%),-(mosaic)(19)(55%),-(mosaic)(21)(50%),-(mosaic)(22)(60%)	Simple aneuploidy
		#2	49,XX,-2,+7,+9,+10,-14,+21,+22	Complex aneuploidy
		#3	46,XX	Euploidy
P43	-	#1	47,XX,-(mosaic)(1)(63%),-2,+(mosaic)(3)(34%),-(mosaic)(4)(53%),+(mosaic)(5)(59%),-(mosaic)(6)(58%),-(mosaic)(9)(65%),dup(10)(p15.3-p11.23)(27.8Mb),+14,+16,+(mosaic)(18)(58%)	Complex aneuploidy
		#2	46,XY,-(mosaic)(5)(44%),dup(mosaic)(8)(q11.21-q21.13)(34.6Mb)(49%),+(mosaic)(13)(33%),+(mosaic)(14)(38%)	Other
P44	-	#1	46,XX	Euploidy
		#2	46,XY	Euploidy
P45	-	#1	45,XY,-13	Simple aneuploidy
		#2	46,XY, +(mosaic)(8)(38%), -(mosaic)(10)(31%), -(mosaic)(16)(41%), -(mosaic)(22)(45%)	Other
P46	-	#1	46,XX,-1,+2,-4,+11,-14,-15,+16,+22,dup(X)(p22.33-q21.1)(73.4Mb),dup(mosaic)(X)(q21.1-q28)(66.2Mb)(59%)	Complex aneuploidy
		#2	51,XY,+(mosaic)(3)(32%),-4,del(mosaic)(5)(q11.2-q35.3)(121.8Mb)(58%),+7,+(mosaic)(8)(32%),del(9)(p24.3-p13.1)(36.4Mb),del(mosaic)(9)(q21.11-q34.3)(68.4Mb)(45%),+10,+13,+14,	Complex aneuploidy

			del(16)(p13.3-p11.2)(26.2Mb),del(mosaic)(16)(q12.1-q24.3)(41.8Mb)(49%),+19,+22	
P47	-	#1	47,XY,+1	Simple aneuploidy
		#2	46,XY,+(mosaic)(8)(41%),+(mosaic)(14)(31%),-(mosaic)(18)(58%),-(mosaic)(21)(32%)	Other
P48	-	#1	45,XY,+2,+(mosaic)(3)(62%),+(mosaic)(4)(34%),-(mosaic)(5)(33%),-(mosaic)(9)(46%),+(mosaic)(11)(40%),-(mosaic)(12)(37%),-(mosaic)(13)(41%),-14,-(mosaic)(16)(46%),-18,+(mosaic)(19)(34%),+(mosaic)(20)(69%),+(mosaic)(21)(30%),+(mosaic)(X)(35%)	Complex aneuploidy
		#2	47,XY,+20	Simple aneuploidy
P49	-	#1	45,XY,-1,+3,-4,-5,-8,+9,+13,+14,-20	Complex aneuploidy
		#2	41,XO,-6,dup(8)(p23.3-q22.3)(98.0Mb),-9,+10,-12,+14,-16,-19,+20,-22,-X	Complex aneuploidy
P50	-	#1	44,XY,+4,-10,-11,-21,-(mosaic)(X)(65%)	Complex aneuploidy
		#2	45,XX,-1,del(mosaic)(2)(q21.3-q37.3)(104.4Mb)(30%), del(mosaic)(6)(q22.1-q27)(54.8Mb)(35%),+13,-16,+(mosaic)(19)(57%),-(mosaic)(21)(46%)	Complex aneuploidy

Supplementary Table S7. Chromosome copy number results of blastulation failure embryos from S-GQBF cohort.

Patients	No. of embryo	Single-embryo-based whole-genome sequencing	Classified
C1	#1	45,XX,-17	Simple aneuploidy
C2	#1	46,XX	Euploidy
	#2	45,XX,-(mosaic)(4)(40%),-(mosaic)(6)(32%),-(mosaic)(8)(32%),-(mosaic)(15)(41%),-17,-(mosaic)(20)(34%)	Simple aneuploidy
C3	#1	45,XY,-(mosaic)(16)(59%),-20	Simple aneuploidy
C4	#1	47,XY,+16	Simple aneuploidy
	#2	46,XX	Euploidy
	#3	46,XY,-(mosaic)(13)(32%)	Other
C5	#1	47,XX,+(mosaic)(13)(57%),del(mosaic)(16)(q12.1-q24.3)(42.48Mb)(39%),+19	Simple aneuploidy
	#2	46,XX,-(mosaic)(8)(31%),-(mosaic)(14)(43%),-(mosaic)(22)(40%)	Other
C6	#1	46,XX	Euploidy
C7	#1	46,XX	Euploidy
	#2	45,XY,-22	Simple aneuploidy
C8	#1	46,XY,dup(mosaic)(4)(q28.1-q35.2)(62.2Mb)(62%)	Other
	#2	46,XY,-(mosaic)(6)(36%),-(mosaic)(10)(34%),-(mosaic)(13)(30%),+(mosaic)(14)(38%), +(mosaic)(19)(31%),+(mosaic)(X)(31%)	Other
C9	#1	46,XX,dup(mosaic)(2)(p24.1-p12)(56.4Mb)(35%),dup(mosaic)(5)(q11.1-q14.3)(39.3Mb)(38%),dup(mosaic)(17)(q23.1-q25.3)(21.0Mb)(40%)	Other
	#2	46,XY	Euploidy

	#3	46,XX,del(mosaic)(10)(q23.33-q26.3)(38.8Mb)(37%),del(X)(q24-q28)(33.0Mb)	Other
C10	#1	46,XY	Euploidy
	#2	46,XY,+(mosaic)(5)(36%)	Other
C11	#1	46,XX,+(mosaic)(19)(47%)	Other
	#2	44,XX,-6,-13,del(X)(p22.31)(2.2Mb)	Simple aneuploidy
C12	#1	46,XX	Euploidy
	#2	46,XY	Euploidy
C13	#1	45,XY,-21	Simple aneuploidy
	#2	46,XY,-15,+21	Simple aneuploidy
C14	#1	45,XY,-2	Simple aneuploidy
C15	#1	45,XY,-21	Simple aneuploidy
	#2	47,XY,+16	Simple aneuploidy
C16	#1	46,XX	Euploidy
	#2	47,XX,+19,+(mosaic)20(37%)	Simple aneuploidy
	#3	46,XX	Euploidy
C17	#1	47,XX,del(11)(q14.1-q25)(49.4Mb),+22	Simple aneuploidy
	#2	47,XY,+1	Simple aneuploidy
C18	#1	45,XY,-15	Simple aneuploidy
	#2	44,XY,-16,-22	Simple aneuploidy
C19	#1	46,XX	Euploidy
	#2	46,XY,-(mosaic)(7)(54%),del(mosaic)(9)(p13.3-q34.3)(71.6Mb)(30%),-(mosaic)(12)(31%),-(mosaic)(13)(45%),+(mosaic)(14)(42%)	Other

C20	#1	45,XY,-16	Simple aneuploidy
	#2	46,XX,dup(mosaic)(3)(p12.3-p12.1)(10.82Mb)(38%)	Other
C21	#1	46,XY,+(mosaic)(1)(42%),-(mosaic)(18)(35%),-(mosaic)(Y)(34%)	Other
	#2	47,XX,-(mosaic)(13)(32%),+22	Simple aneuploidy
C22	#1	46,XX,+(mosaic)(10)(46%),-(mosaic)(14)(58%),+(mosaic)(X)(31%)	Other
C23	#1	45,XY,-9	Simple aneuploidy
	#2	47,XX,+2,-(mosaic)(16)(52%),-(mosaic)(21)(56%),-(mosaic)(22)(54%)	Simple aneuploidy
C24	#1	46,XX	Euploidy
	#2	46,XY,+(mosaic)(16)(33%),-(mosaic)(20)(45%)	Other
C25	#1	39,XY,-1,-2,-3,-4,-9,-14,-21	Complex aneuploidy
	#2	46,XY	Euploidy
	#3	46,XY	Euploidy
C26	#1	47,XY,+13	Simple aneuploidy
	#2	44,XY,-7,-13	Simple aneuploidy
C27	#1	46,XX,+(mosaic)(8)(33%),-(mosaic)(16)(53%)	Other
	#2	46,XX,del(mosaic)(8)(q21.3-q24.3)(54.2Mb)(32%)	Other
C28	#1	46,XX,del(4)(q31.1-q35.2)(48.6Mb),del(mosaic)(9)(q22.1-q34.3)(50.0Mb)(41%)	Other
	#2	46,XY,+(mosaic)(1)(45%),dup(mosaic)(3)(q12.1-q24)(46.4Mb)(35%),-(mosaic)(5)(36%),-(mosaic)(8)(44%),+(mosaic)(10)(37%),-(mosaic)(17)(65%),+(mosaic)(18)(51%),-(mosaic)(19)(35%),+(mosaic)(21)(34%),-(mosaic)(22)(41%)	Other
C29	#1	46,XY,-17,+22	Simple aneuploidy
C30	#1	47,XX,+16	Simple aneuploidy

#2	46,XX,+(mosaic)(5)(53%)	Other

Supplementary Table S8. Sperm disomy, nullisomy, and diploidy rates in individuals with *MEIKIN* variants and a proven fertile control (free of *MEIKIN* variants).

Chr	Control (%, N/n)			Family 18 (%, N/n)			Family 19 (%, N/n)			Family 20 (%, N/n)		
	nullisomy rate	disomy rate	diploidy rate	nullisomy rate	disomy rate	diploidy rate	nullisomy rate	disomy rate	diploidy rate	nullisomy rate	disomy rate	diploidy rate
1	0 (0/1228)	0.16 (2/1228)		25.93 (112/432)	15.74 (68/432)		17.67 (208/1177)	12.23 (144/1177)		3.54 (39/1101)	6.27 (69/1101)	
6	0.08 (1/1228)	0.08 (1/1228)	0 (0/1228)	26.16 (113/432)	16.90 (73/432)	3.01 (13/432)	8.16 (96/1177)	20.14 (237/1177)	5.52 (65/1177)	1.91 (21/1101)	10.90 (120/1101)	5.36 (59/1101)
9	0.16 (2/1228)	0 (0/1228)		13.19 (57/432)	17.82 (77/432)		14.61 (172/1177)	12.40 (146/1177)		1.36 (15/1101)	9.26 (102/1101)	
2	0.15 (2/1311)	0.08 (1/1311)		9.02 (49/543)	12.71 (69/543)		17.08 (193/1130)	20.53 (232/1130)		3.98 (45/1130)	6.37 (72/1130)	
4	0.08 (1/1311)	0.08 (1/1311)	0 (0/1311)	10.31 (56/543)	16.57 (90/543)	2.58 (14/543)	20.62 (233/1130)	17.70 (200/1130)	7.61 (86/1130)	1.86 (21/1130)	10.80 (122/1130)	5.04 (57/1130)
12	0 (0/1311)	0.15 (2/1311)		9.02 (49/543)	12.71 (69/543)		14.60 (165/1130)	22.57 (255/1130)		1.68 (19/1130)	9.47 (107/1130)	
3	0.08 (1/1328)	0.08 (1/1328)		14.35 (65/453)	8.83 (40/453)		18.82 (220/1169)	22.07 (258/1169)		2.56 (27/1056)	10.98 (116/1056)	
7	0.08 (1/1328)	0.08 (1/1328)	0.08 (1/1328)	23.40 (106/453)	7.51 (34/453)	0.88 (4/453)	16.51 (193/1169)	19.25 (225/1169)	5.05 (59/1169)	1.04 (11/1056)	10.98 (116/1056)	6.44 (68/1056)
15	0.08 (1/1328)	0.08 (1/1328)		5.08 (23/453)	10.15 (46/453)		15.57 (182/1169)	16.17 (189/1169)		2.65 (28/1056)	10.13 (107/1056)	
18	0.08 (1/1241)	0.16 (2/1241)	0 (0/1241)	20.00 (64/320)	14.69 (47/320)	5.31 (17/320)	16.68 (156/935)	31.98 (299/935)	15.08 (141/935)	1.66 (19/1145)	8.30 (95/1145)	6.64 (76/1145)
XY	0.08 (1/1241)	0.16 (2/1241)		4.69 (15/320)	12.50 (40/320)		13.48 (126/935)	25.78 (241/935)		1.14 (13/1145)	9.00 (103/1145)	
Total	0.87	1.11	0.08	134.99	146.13	11.78	173.8	220.82	33.26	23.38	102.46	23.48

.

Supplementary Table S9. Single-sperm whole-genome sequencing in individuals carrying *MEIKIN* variants and a proven fertile control (free of *MEIKIN* variants).

Family	Size of sperm head	No. of sperm	Sequencing type	Single-sperm-based whole-genome sequencing
Family 18	Normal	#1	Low-depth WGS	X, +6, -8, -11, -12, -13, -14, -15, -16, +18, -19, +20
Family 18	Normal	#2	Low-depth WGS	XX, +1, -2, -3, +6, +7, -8, +13, +16, -22
Family 18	Normal	#3	Low-depth WGS	YY, -1, -2, +6, +7, +9, -10, -12, -13, +15, -19, +20, -21, -22
Family 18	Normal	#4	Low-depth WGS	YY, +2, +3, +11, -12, -16, -19
Family 18	Normal	#5	Low-depth WGS	X, -1, +3, +7, +8, -9, +12, -14, -16, +18, +20, -21, +22
Family 18	Normal	#6	Low-depth WGS	X, +9, +10, +11, -13, -14, -15, +16, +18
Family 18	Normal	#7	Low-depth WGS	XX, -2, -7, -8, -10, -15, +16, -21
Family 18	Large	#1	Low-depth WGS, HaploPGT	X, Haploidy? → XX, Diploidy
Family 18	Large	#2	Low-depth WGS, HaploPGT	Y, Haploidy? → YY, Diploidy
Family 18	Large	#3	Low-depth WGS, HaploPGT	Y, Haploidy? → YY, Diploidy
Family 18	Large	#4	Low-depth WGS, HaploPGT	Y, Haploidy? → YY, Diploidy
Family 18	Large	#5	Low-depth WGS, HaploPGT	Y, Haploidy? → YY, Diploidy
Family 18	Small	#1	Low-depth WGS	X, -1, +3, +5, +7, +8, +10, +11, +12, +13, -14, +15, -16, +19, -22
Family 18	Small	#2	Low-depth WGS	Sex chromosome loss, -2, -3, -4, +5, -7, -9, -11, -15, +16, +17, -18, -21
Family 18	Small	#3	Low-depth WGS	YY, +1, +2, -10, +14, +15, +19, +20

Family 18	Small	#4	Low-depth WGS	Sex chromosome loss, -1, +5, +7, -11, +13, -14, -15, -17, -19, -20, -21, -22
Family 18	Small	#5	Low-depth WGS	X, +3, -8, -9, +13, -14, -16, +17, +19, +22
Family 19	Normal	#1	Low-depth WGS	X, +6, -7, +8, -9, -10, -11, -14, -18, +19, -20, +21
Family 19	Normal	#2	Low-depth WGS	Y, -2, +7, -9, -10, -12, -14, -17, -20
Family 19	Normal	#3	Low-depth WGS	X, -1, +3, -11, -12, +13, +15, -22
Family 19	Normal	#4	Low-depth WGS	YY, -2, +3, -5, -9, +14, +16, -17, +18, -19, +21, +22
Family 19	Normal	#5	Low-depth WGS	Y, +3, -4, -9, +10, +11, +13, -14, -16, -17, +19, -21
Family 19	Normal	#6	Low-depth WGS	Y, -1, +2, -3, -5, +9, +11, -14, -15, -17, +18, -20
Family 19	Normal	#7	Low-depth WGS	Y, +1, +3, -4, -7, -10, +11, +12, +13, -14, -15, -16, -21
Family 19	Normal	#8	Low-depth WGS	Y, -6, +8, -11, -12, -13, -14, +16, -17, -18, -20, -21
Family 19	Normal	#9	Low-depth WGS	YY, -1, +3, -4, +5, +12, -14, -16, -17, -18, -22
Family 19	Normal	#10	Low-depth WGS	X, -1, +2, -3, -4, +5, +6, -7, +9, -10, +11, +12, +14, -15, +18, -19, +20, -22
Family 19	Normal	#11	Low-depth WGS	YY, -1, -4, -7, -15, +17, +19, -22
Family 19	Normal	#12	Low-depth WGS	Y, -1, -3, +5, -6, -8, +9, +10, -11, -12, +13, +14, +19, -21
Family 19	Normal	#13	Low-depth WGS	Y, -1, +8, -13, -14, -15, +16, +21
Family 19	Normal	#14	Low-depth WGS	Sex chromosome loss, -2, -3, -5, +7, -8, +9, -10, -11, +13, +14, +15, -17, +18, -21
Family 19	Normal	#15	Low-depth WGS	X, -2, +3, +4, -11, -13, -14, -16, +17, -18, +21
Family 19	Normal	#16	Low-depth WGS	Y, +4, -5, -6, -9, -10, +12, +13, -14, -17, -18, +20, -21
Family 19	Normal	#17	Low-depth WGS	Y, +7, +8, +9, -14, -19, -20, +22
Family 19	Normal	#18	Low-depth WGS	Y, -4, +5, -6, -7, -9, -10, -19, -22
Family 19	Normal	#19	Low-depth WGS	X, -2, -3, +8, -9, -12, -13, -14, +15, -18
Family 19	Normal	#20	HaploPGT	X, -4, -6, -9, -12, +13, -18, -19, +20, -21, +22
Family 19	Normal	#21	HaploPGT	Sex chromosome loss, -2, -4, -5, +6, +7, -15, -16, +21

Family 19	Large	#1	HaploPGT	Y, Diploidy
Family 19	Large	#2	HaploPGT	XX, Diploidy
Family 19	Large	#3	HaploPGT	Y, Diploidy
Family 19	Large	#4	HaploPGT	XX, Diploidy
Family 19	Small	#1	Low-depth WGS	Y, +3, -4, -6, +8, -12, -14, +15, -16, -18, -19, -21
Family 19	Small	#2	Low-depth WGS	Y, -1, -2, +3, +4, -8, -10, +11, -13, +15, -17, -19
Family 19	Small	#3	Low-depth WGS	X, +1, +2, -3, -5, -6, -7, -9, +13, +14, -19, -20, +22
Family 19	Small	#4	Low-depth WGS	Sex chromosome loss, +3, -6, -9, +10, -11, +12, -14, -16, +19, +21, -22
Family 19	Small	#5	Low-depth WGS	X, -1, +2, -3, -5, +6, -7, -9, +10, +11, +13, +14, +16, +17, +19
Family 19	Small	#6	Low-depth WGS	XX, +6, -12, +14, +15, -16, -20, -22
Family 19	Small	#7	Low-depth WGS	X, +1, -5, -9, +10, +11, -12, -14
Family 19	Small	#8	Low-depth WGS	Sex chromosome loss, -1, -4, +9, +10, -13, -15, -16, -21
Family 19	Small	#9	Low-depth WGS	Sex chromosome loss, -1, -2, +5, -6, -7, -10, +11, +15, -16, -18, +19, -20, -21
Family 19	Small	#10	HaploPGT	X, -2, -3, -8, -9, +12, -13, -14, -15, -17, +18, +20, +22
Family 19	Small	#11	HaploPGT	X, -2, -5, +7, -8, +9, +14, +19, +20
Family 20	Normal	#1	Low-depth WGS	Y, -13
Family 20	Normal	#2	Low-depth WGS	Y, -4
Family 20	Normal	#3	Low-depth WGS	Y, -11
Family 20	Normal	#4	Low-depth WGS	Y, -2
Family 20	Normal	#5	Low-depth WGS	Y, +4, -17, -21
Family 20	Normal	#6	Low-depth WGS, HaploPGT	X, Haploidy? → X, Haploidy
Family 20	Normal	#7	Low-depth WGS,	X, Haploidy? → X, Haploidy

			HaploPGT	
Family 20	Large	#1	Low-depth WGS	XY, 10*1, 11*3, 13*3, 15*3, 16*3, presumed to be diploidy
Family 20	Large	#2	Low-depth WGS, HaploPGT	X, Haploidy? → XX, Diploidy
Family 20	Large	#3	Low-depth WGS, HaploPGT	Y, Haploidy? →YY, Diploidy
Family 20	Large	#4	Low-depth WGS, HaploPGT	Y, Haploidy? → YY, Diploidy
Family 20	Small	#1	Low-depth WGS	X, +8, -10, -11, -13, +15, -17, -20
Family 20	Small	#2	Low-depth WGS	Y, +2, -4, +5, +7, +12, +13, +15, +16, +20, +21
Family 20	Small	#3	Low-depth WGS	Sex chromosome loss, +1, -2, +5, +12, +13, -16, -19, +22
Family 20	Small	#4	Low-depth WGS	X, -10, +17
Family 20	Small	#5	Low-depth WGS	X, -3, -4, -7, -10, +19, -20
Control	Normal	#1	Low-depth WGS	Y, Haploidy
Control	Normal	#2	Low-depth WGS	X, Haploidy
Control	Normal	#3	Low-depth WGS	Y, Haploidy
Control	Normal	#4	Low-depth WGS	X, Haploidy
Control	Normal	#5	Low-depth WGS	X, Haploidy
Control	Normal	#6	Low-depth WGS	Y, Haploidy
Control	Normal	#7	Low-depth WGS	X, Haploidy
Control	Normal	#8	Low-depth WGS	X, Haploidy
Control	Normal	#9	Low-depth WGS	X, Haploidy
